# Mobile Text Messaging for Tobacco Risk Communication Among Young Adult Community College Students: Randomized Trial of Project Debunk

**DOI:** 10.2196/25618

**Published:** 2021-11-24

**Authors:** Alexander V Prokhorov, Karen Sue Calabro, Ashish Arya, Sophia Russell, Katarzyna W Czerniak, Gabrielle C Botello, Minxing Chen, Ying Yuan, Adriana Perez, Damon J Vidrine, Cheryl L Perry, Georges Elias Khalil

**Affiliations:** 1 Department of Behavioral Science MD Anderson Cancer Center The University of Texas Houston, TX United States; 2 Department of Health Disparities MD Anderson Cancer Center The University of Texas Houston, TX United States; 3 Department of Health Services Research MD Anderson Cancer Center The University of Texas Houston, TX United States; 4 Department of Biostatistics MD Anderson Cancer Center The University of Texas Houston, TX United States; 5 Department of Biostatistics and Data Science School of Public Health University of Texas Health Science Center Austin, TX United States; 6 Department of Health Outcomes and Behavior Moffitt Cancer Center Tampa, FL United States; 7 School of Public Health Health Science Center University of Texas Austin, TX United States; 8 Department of Health Outcomes and Biomedical Informatics College of Medicine University of Florida Gainesville, FL United States

**Keywords:** tobacco use, risk communication, text messaging, message framing, regulatory science, young adults, vaping, mobile phone

## Abstract

**Background:**

The use of new and emerging tobacco products (NETPs) and conventional tobacco products (CTPs) has been linked to several alarming medical conditions among young adults (YAs). Considering that 96% of YAs own mobile phones, SMS text messaging may be an effective strategy for tobacco risk communication.

**Objective:**

Project Debunk is a community-based randomized trial aiming to identify specific types of messages that effectively improve perceived NETP and CTP risk among YAs in community colleges.

**Methods:**

With YAs recruited offline from 3 campuses at the Houston Community College (September 2016 to July 2017), we conducted a 6-month randomized trial with 8 arms based on the combination of 3 message categories: framing (gain-framed vs loss-framed), depth (simple vs complex), and appeal (emotional vs rational). Participants received fully automated web-based SMS text messages in two 30-day campaigns (2 messages per day). We conducted repeated-measures mixed-effect models stratified by message type received, predicting perceived CTP and NETP risks. Owing to multiple testing with 7 models, an association was deemed significant for *P*<.007 (.05 divided by 7).

**Results:**

A total of 636 participants completed the baseline survey, were randomized to 1 of 8 conditions (between 73 and 86 participants per condition), and received messages from both campaigns. By the 2-month post campaign 2 assessment point, 70.1% (446/636) completed all outcome measures. By the end of both campaigns, participants had a significant increase in perceived NETP risk over time (*P*<.001); however, participants had a marginal increase in perceived CTP risk (*P*=.008). Separately for each group, there was a significant increase in perceived NETP risk among participants who received rational messages (*P*=.005), those who received emotional messages (*P*=.006), those who received simple messages (*P*=.003), and those who received gain-framed messages (*P*=.003).

**Conclusions:**

In this trial, YAs had an increase in perceived NETP risk. However, with stratification, we observed a significant increase in perceived NETP risk upon exposure to rational, emotional, simple, and gain-framed messages. In addition, YAs generally had an increase in perceived CTP risk and presented nonsignificant but observable improvement upon exposure to emotional, complex, and loss-framed messages. With the results of this study, researchers and practitioners implementing mobile health programs may take advantage of our tailored messages through larger technology-based programs such as smartphone apps and social media campaigns.

**Trial Registration:**

ClinicalTrials.gov NCT03457480; https://clinicaltrials.gov/ct2/show/NCT03457480

**International Registered Report Identifier (IRRID):**

RR2-10.2196/10977

## Introduction

### Background

Tobacco use in many forms, such as combustible, vaporized, or smokeless, has been linked to several alarming medical conditions among young adults (YAs; aged 18-25 years). These include nicotine dependence [1], psychiatric disorders [2], and developing pulmonary [3,4] and cardiovascular diseases [5]. Of more recent concern are the findings of deleterious health effects associated with inhalation of nicotine-containing aerosol (vaping) [6,7] and longitudinal associations between vaping and future use of conventional tobacco products (CTPs; including combustible cigarettes, cigars, pipes, and chewing tobacco, dip, or snuff) [8-11]. However, approximately 15% of American YAs are current cigarette smokers, and 36% of American YAs are current users of vaping products, one of many new and emerging tobacco products (NETPs), including vapes, hookahs, and snus in the United States [12].

YA tobacco use can be partly attributed to the relatively low perceived risk of products when compared with other adult age groups [13-15]. This is particularly the case for NETPs such as vaping products and hookahs, which are believed to be safer than CTPs [13,16-18]. According to national reports, YAs tend to have a lower perception of the harm from vaping products [19] and hookah [20] compared with combustible cigarettes. YAs tend to demonstrate a lack of knowledge about the ingredients in vaping products [14,21,22]. Ultimately, lower risk perception, among other factors, has led to health-compromising behaviors among YAs [23], including experimentation with various nicotine and tobacco products as well as other substances [24-26].

### Responding to Tobacco Marketing

Despite known health consequences, aggressive tobacco marketing to young people has been found to reduce risk perception and promote continued tobacco use [27,28]. YAs form a highly vulnerable population that continues to be targeted as a potentially profitable market segment for the tobacco industry [27-30]. The tobacco industry broadly disseminates modern advertising on the radio, television, the internet, in print, through direct mail, in nightclubs and pubs, and at the point of sale [31-36].

Tobacco advertising to YAs has become particularly successful through mobile media channels [37,38]. Today, tobacco companies depend on mobile strategies for marketing, considering that 96% of YAs own smartphone devices [39]. Mobile marketing forums constitute the next generation of marketing strategies, notably with demonstrations and invitations through social media websites [40-43] and a variety of protobacco smartphone apps advertised under *kids* and *games* categories [44].

Health promotion experts and activists ought to respond to tobacco marketing by communicating tobacco risk to YAs as delineated by the educational mission and research priorities of the US Food and Drug Administration [45,46]. Considering that nearly all YAs (96%) own mobile phones, mobile phone SMS text messaging is likely to be an effective strategy for tobacco risk communication [39,47]. Although YAs tend to use a variety of mobile phone apps for communication (eg, WhatsApp), SMS text messaging remains a universal and practical method of risk communication. SMS text messaging programs have been successfully implemented for preventive behavioral treatment, including smoking cessation [48]. However, there have been no published accounts for its application in communicating tobacco risk to YAs [49-53].

### Project Debunk: A Text Messaging Program

The goal of our project (Project Debunk) was to develop a library of risk communication messages. Our message design was based on a combination of 3 main message categories, each with 2 message types: (1) framing (gain-framed or loss-framed messages), (2) depth (ie, simple or complex messages), and (3) appeal (ie, emotional or rational messages). Framing and appeal were supported by previous research [54-57], whereas depth was an original category proposed by our investigative team. Messages from each category were also developed to communicate the harm of CTPs and NETPs (Multimedia Appendix 1). Messages describing CTPs included information regarding combustible cigarettes, cigars, and pipes. Messages describing NETPs included information regarding vaping devices, snus, little cigars, cigarillos, and hookah (products that were becoming increasingly prevalent at the time of this study [12]). The resulting 976 SMS text messages were designed through focus group discussions with YAs and feedback from experts in public health, health communication, and behavioral science [58]. The results indicate that YAs find the messages interesting and appropriate. They described the messages as informative, interesting, easy to understand, straight to the point, and at an appropriate character limit [58]. As a result, these messages are of interest to YAs who would like to be informed regarding tobacco. These SMS text messages have been validated using linguistic inquiry and word counting, indicating an adequate design based on framing, depth, and appeal [59]. In addition, early analysis from a Project Debunk randomized trial further validated the messages [60]. Loss-framed messages were more likely to be perceived as presenting a loss than gain-framed messages, complex messages were reported to be more complex than simple messages, and emotional messages were perceived to be more emotionally engaging than rational messages [60]. In addition, there were no differences among the message types with respect to reported message credibility, message enjoyment, perceived message relevance, or message readability level [60]. A detailed description of the trial and baseline characteristics has been published under the study protocol [60].

### Theoretical Framework

Available theoretical frameworks have described that the success of message characteristics depends on individual differences in the way they process information. First, according to the elaboration likelihood model (ELM) [61,62], the effectiveness of message characteristics depends on one’s cognitive effort used to engage with the message content. When centrally processing information, individuals put more effort into paying attention to message content (eg, complex and rational messages [63]). On the other hand, when peripherally processing information, individuals put less cognitive effort by paying attention to more peripheral cues, such as emotional features of the message [64]. Second, according to the prospect theory of message framing, gain and loss framing can have an effect on health behavior depending on whether the individual is risk-aversive or risk-taking [65]. Regardless, different message characteristics can be effective for different audience members. Results from previous meta-analyses of relevant research have not favored one message type over another when improving health outcomes [66-68]. As a result, it is essential to explore the effects of different message characteristics on perceived risk.

### Study Objective

The objective of this paper on Project Debunk is to present the results of a community-based randomized trial. The trial aims to identify specific types of messages that are effective in increasing the perceived risk of NETP use and CTP use among YAs in community colleges. Considering the limited research, we cannot predict or anticipate differences among message types in improving perceived tobacco risk [60]. For this reason, we will test the success of improving perceived risk over time among participants exposed to each message type alone. Our central hypothesis is that controlling for all other message types, campaign participants receiving each message type will have an increase in perceived NETP risk and perceived CTP risk over time. A message type is deemed impactful if it improves YAs’ perceived risk over time. Once impactful SMS text messages have been identified, they can subsequently be introduced into an advanced digital intervention.

## Methods

### Study Design

A detailed description of the study design has been presented elsewhere [60]. Briefly, we conducted a 6-month randomized trial (September 2016 to July 2017) with 8 arms based on a combination of 3 message categories: framing, depth, and appeal (NCT03457480). Each category included 2 message types, leading to a 2 (framing: gain vs loss) ×2 (depth: simple vs complex) ×2 (appeal: rational vs emotional) factorial design. Randomization in this design allowed us to control for receiving different message types, as we tested changes over time for each message type. Our objective was to examine changes in perceived risk over time. As a result, comparison with a control group was not conducted.

Participants received SMS text messages on their mobile phones for free in 2 waves or campaigns. Each campaign comprised 2 SMS text messages per day for 30 days (ie, 60 text messages). This resulted in a total of 120 messages considering both campaigns. Participants were randomized into 8 arms, with 8 permutations based on message types. As a result, there was a total of 960 messages disseminated for this study. The 2 campaigns were performed 1 week apart. The development process and content of messages were *frozen* during the trial.

**Figure 1:**
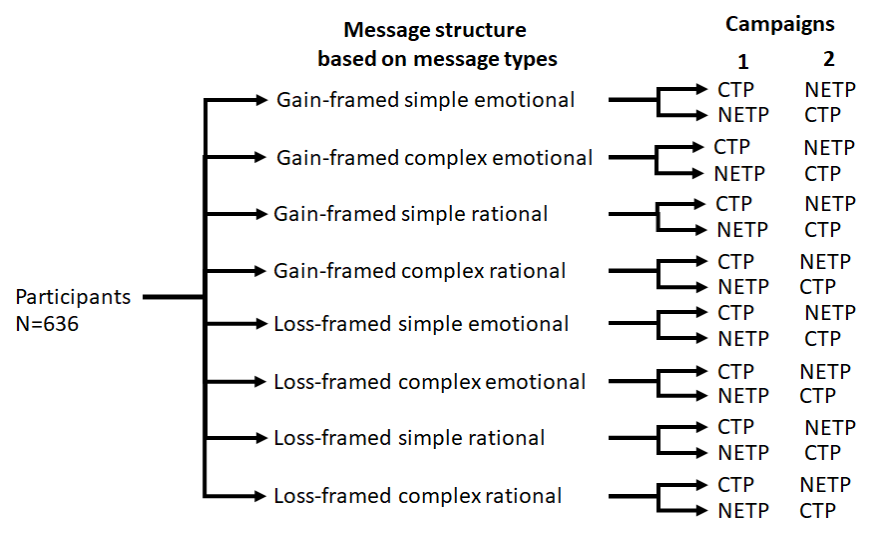
Study design and randomization to 8 conditions followed by 2 crossover conditions (total of 16 conditions). CTP: conventional tobacco product; NETP: new and emerging tobacco product; G: gain-framed messages, L: loss-framed messages, C: complex messages, S: simple messages, R: rational messages, and E: emotional messages.

For ethical reasons, all participants received both NETP and CTP messages. To control for the order in which SMS text messages were received, the study included a crossover design. Participants within each arm were randomly divided into 2 groups: the first group received messages on CTPs during the first campaign and then NETPs during the second campaign, whereas the second group received messages on NETPs during the first campaign and then CTPs during the second campaign (Figure 1). The study adhered to the CONSORT (Consolidated Standards of Reporting Trials) and CONSORT-EHEALTH (CONSORT–Electronic and Mobile Health Applications and Online TeleHealth) guidelines (Multimedia Appendix 2) [69,70].

### Population

YAs were recruited from 3 campuses at the Houston Community College. The campuses were selected based on their ethnically diverse populations [71]. YAs were eligible for the study if they were aged between 18 and 25 years, enrolled in a community college, possessed a mobile phone, regularly used SMS text messaging, were willing to provide their phone number, were capable of receiving SMS text messages from our messaging system, and were able to read and speak English. The study protocol was approved by the institutional review boards of the University of Texas MD Anderson Cancer Center and Houston Community College.

### Recruitment and Enrollment

At each of the 3 campuses, recruitment took place face to face at highly visible recruitment stations or booths, and printed materials announcing the study (posters and fliers) were displayed in high traffic areas. Research staff screened interested students for eligibility, and eligible students provided informed consent to participate in the study. During the face-to-face consent process, participants received information regarding the objective of the study, study procedures, potential risks, potential benefits, compensation information, and contact information. Following consent, participants completed a 20-minute web-based baseline survey on their phones. Participants received a US $25 gift card for completing the baseline survey and each of the 2 postcampaign surveys.

After 3 days of receiving the baseline survey, YAs began to receive SMS text messages through the MD Anderson Cancer Center resource called assessment, intervention, and measurement. A password-protected allocation sequence was generated by the assessment, intervention, and measurement resource, automatically sending SMS text messages on the basis of allocation and keeping the research team blind to participant allocation. Participants were blinded to the type of message they received. Research assistants were available over the phone in case of usability issues.

### Measures

Through web-based skip-pattern surveys, we assessed a series of previously validated and pretested measures [72]. The measures have been validated for web-based use. We listed all measures within our research protocol for the current trial [60] and adhered to the Checklist for Reporting Results of Internet E-Surveys (previously published as a supplementary material [60]). At baseline, participants provided information regarding their age, gender, race, ethnic group, basic expenses, education attainment, numeracy level [73], and current use of CTP and NETP (ie, past 30 days) [74,75]. Immediately after each campaign, we assessed the self-reported attention level to the messages (2 items, such as *“*When I was reading the text messages, I paid attention to the messages more than to what was happening around me.”). Participants were asked if this was true for none of the messages (1), 1-2 messages (2), some of the messages (3), a lot of the messages (4), or all the messages (5). At baseline, 2 months post campaign 1, and 2 months post campaign 2, we assessed perceived CTP risk and perceived NETP risk [76]. Using a validated scale [76], the perceived risk of using cigarettes, cigars, little cigars, cigarillos, pipes, chewing tobacco, and dip or snuff, hookah, vaping products, and snus was measured separately for each product. This allowed respondents to distinguish between the risks of different tobacco products. The measure included 5 items; with the first item on a 4-point Likert scale from no risk to great risk, we asked respondents how much they think people risk harming themselves if they use each of the tobacco products. On a 4-point Likert scale from strongly disagree to strongly agree, the 4 remaining items presented statements such as, “The following products increase the risk for medical problems such as reproductive problems, respiratory problems, or heart disease” [76]. Depending on product type, Cronbach α scores ranged between .72 and .87. Perceived NETP risk was measured as the average score for vaping products, hookah, little cigars or cigarillos, and snus. Perceived CTP risk was measured as the average score for cigarettes, cigars, pipes, and chewing tobacco, dip, or snuff. The use of hookah, little cigars, or cigarillos, snus, and electronic cigarettes became increasingly prevalent at the time of this study, making such products new and emerging [12]. This was particularly the case in Texas [77]. For this reason, we chose to treat them as NETPs.

### Statistical Analysis

Sample size determination has been previously described [60]. First, chi-square tests and 1-way analyses of variance were conducted to check for differences among groups with respect to the digital divide (ie, gaps in access to mobile phones), confounders, and message reception. Then, we examined 2 study outcomes (perceived CTP risk and perceived NETP risk) and their change over time (from baseline to 2 months post campaign 2). To test the overall campaign success, we conducted 2 repeated-measures mixed-effect models for all participants, examining changes over time in perceived CTP risk and perceived NETP risk. This pair of models included the time effect and main effects of message types: assignment to gain-framed messages, assignment to emotional messages, and assignment to simple messages.

Our central hypothesis is that, controlling for all other message types, campaign participants receiving each message type will have an increase in perceived NETP risk and perceived CTP risk over time. To test the success of improving perceived risk over time among participants exposed to specific message types, 6 models were used for participants receiving (1) rational messages, (2) emotional messages, (3) complex messages, (4) simple messages, (5) loss-framed messages, and (6) gain-framed messages. These 6 models were conducted to predict perceived CTP risk and then perceived NETP risk. All 7 models that predicted each main outcome controlled for crossover group assignment and the differential effect of crossover group assignment on time. In addition, after examining potential covariates through a series of regression analyses, all models controlled for age, gender, having a child, basic expenses, education plan, numeracy level, and past 30-day tobacco use at baseline (Multimedia Appendix 3). All models were fitted with restricted or residual maximum likelihood estimation. Considering 7 models for each main outcome, a *P* value <.007 (.05 divided by 7) was considered significant, and a *P* value <.008 was considered marginal [78-81]. We also report the direction of relationships for predictions with *P*<.05 when they are concerned with our study aim. We used STATA (version 14; StataCorp LLC) for our analyses.

## Results

### Attrition

**Figure 2:**
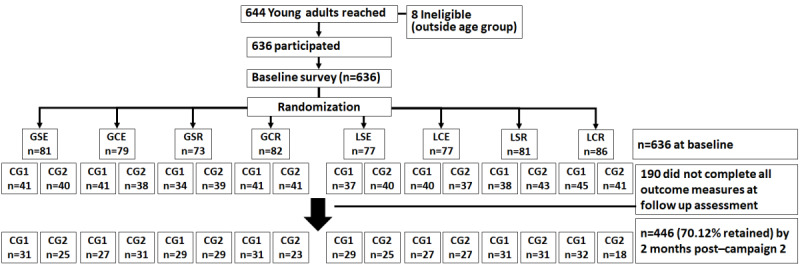
Study flow diagram. CG: crossover group; CG1 receive messages about new and emerging tobacco products (NETP) during campaign 1 and then messages about conventional tobacco product (CTP) during campaign 2; CG2 receive messages about CTP during campaign 1 and then messages about NETP during campaign 2; GCE: gain-framed, complex, emotional; GCR: gain-framed, complex, rational; GSE: gain-framed, simple, emotional; GSR: gain-framed, simple, rational; LCE: loss-framed, complex, emotional; LSE: loss-framed, simple, emotional; LCR: loss-framed, complex, rational; LSR: loss-framed, simple, rational.

Figure 2 presents the study flow diagram. Of the 644 YAs who agreed to participate, we excluded 8 (1.2%) YAs who did not meet the age criterion (aged > 25 years). All 636 participants completed the baseline survey, were randomized to 1 of the 16 conditions, and received the SMS text messages of campaign 1 as prescribed. All participants continued until 2 months post campaign 2; however, 29.9% (190/636) did not complete all outcome measures at the 2-month post campaign 2 assessment (70.1% completion rate).

### Demographic Characteristics

Demographic characteristics for the entire sample and stratified by group at baseline were described under the trial protocol [60]. In summary, the average age of our study sample was 20.78 (SD 2.18) years, and approximately two-thirds of participants (430/636, 67.6%) were male. Participants exhibited a numeracy level of 4.75 (SD 1.87) out of 8, and 29.1% (185/636) aimed to continue their education to receive a doctorate degree. In addition, 35.1% (223/636) reported that they *just meet* basic expenses. The perceived CTP risk for the sample at baseline was 2.56 (SD 0.69), and it became 2.64 (SD 0.71) out of 3 at follow-up. The perceived NETP risk for the sample at baseline was 2.16 (SD 0.77), and it became 2.41 (SD 0.76) out of 3 at follow-up. Multimedia Appendix 4 presents the demographic characteristics of the sample by group.

G stands for gain-framed, L stands for loss-framed, C stands for complex, S stands for simple message, R stands for rational, and E stands for emotional. CG1 stands for crossover group 1 (receiving messages about NETPs during campaign 1 and then messages about CTPs during campaign 2). CG2 stands for crossover group 2 (receiving messages about CTPs during campaign 1 and then messages about NETPs during campaign 2). Retention is based on completing all survey questions pertaining to perceived NETP and CTP risk.

### Checking for the Digital Divide

The results indicate that the groups did not differ with respect to their preferred method of communication (*χ*^2^_7_=22.1; *P*=.07), willingness to receive health SMS text messages (*χ*^2^_7_=5.9; *P*=.56), or frequency of carrying a phone (*χ*^2^_7_=3.4; *P*=.84).

### Message Reception

A series of 1-way analyses of variance indicated no significant difference in attention scores between emotional and rational messages (*F*_1,421_<0.001; *P*=.99), simple and complex messages (*F*_1,421_=1.09; *P*=.29), and gain-framed and loss-framed messages (*F*_1,421_=0.86; *P*=.35). Only approximately 2.8% (12/423) of participants indicated not paying attention to any of the messages. The average score for attention to the messages (mean 3.16, SD 0.93) was significantly higher than 3 out of 5, indicating paying attention to more than *some of the messages* (*P*<.001).

### Overall Campaign Outcomes

Table 1 presents the overall changes in the main outcomes over time. By the end of both campaigns, as indicated by the coefficient for time, participants had a significant increase in perceived NETP risk (*P*<.001; Table 1). In this model, although not significant, NETP users generally exhibited lower scores in perceived NETP risk compared with nonusers (*P*=.009). By the end of both campaigns, participants had a marginal increase in perceived CTP risk (*P*=.008; Table 1).

**Table 1:** Change over time in perceived risk of using NETP^a^ and CTP^b^ for the sample (N=636).^c^

Characteristics			Perceived NETP risk					Perceived CTP risk		
			B (SE)		*P* value		B (SE)			*P* value
Time^d^			0.23 (0.06)		<.001		0.13 (0.05)			.008
Crossover group			−0.04 (0.06)		.49		−0.04 (0.05)			.45
Crossover group by time			0.02 (0.08)		.79		0.05 (0.07)			.50
Age			0.02 (0.01)		.08		−0.001 (0.01)			.93
Being female			−0.01 (0.06)		.78		0.01 (0.05)			.77
Having a child			−0.05 (0.09)		.55		0.09 (0.08)			.23
**Basic expenses**										
	Just meet	0.08 (0.10)		.44		0.08 (0.09)			.37	
	Meet adequately	0.14 (0.11)		.19		0.17 (0.09)			.06	
	Meet comfortably	0.10 (0.11)		.38		0.02 (0.09)			.84	
	Cannot meet (reference)	—^e^		—		—			—	
**Education plan**										
	Associate degree	−0.09 (0.14)		.52		0.12 (0.12)			.33	
	Bachelor’s degree	−0.14 (0.13)		.28		0.003 (0.10)			.19	
	Master’s degree	−0.11 (0.13)		.38		0.06 (0.10)			.08	
	Doctorate degree	−0.09 (0.13)		.49		0.16 (0.10)			.13	
	Certificate (reference)	—		—		—			—	
Numeracy level			0.01 (0.02)		.48		0.01 (0.12)			.28
Baseline use of NETP^f^			−0.16 (0.06)		.009		—			—
Baseline use of CTP^f^			—		—		−0.10 (0.06)			.10
Receive gain-framed messages^g^			0.10 (0.05)		.05		0.05 (0.04)			.21
Receive emotional messages^g^			0.01 (0.05)		.84		0.02 (0.04)			.57
Receive simple messages^g^			0.13 (0.05)		.01		0.07 (0.04)			.11

^a^NETP: new and emerging tobacco product.

^b^CTP: conventional tobacco product.

^c^Two models are presented in this table. Unstandardized coefficients are presented, and the significance level is examined at .007. Multimedia Appendix 5 presents 95% CIs for each coefficient.

^d^The unadjusted time effect predicting perceived NETP risk was B (SE)=0.24 (0.04), *P*<.001, and the unadjusted time effect predicting perceived CTP risk was B (SE)=0.16 (0.03), *P*<.001.

^e^For reference factors (eg, cannot meet), this indicates that data is not applicable. For actual variables (eg, baseline NETP use), this indicates that the variable was not included in the model.

^f^Baseline use of NETP or CTP indicates past 30-day use of NETPs and CTPs at baseline.

^g^These variables compare receiving 1 message type with its counterpart (gain-framed vs loss-framed, emotional vs rational, and simple vs complex).

### Checking for Confounders

To check for potential demographic confounders of perceived CTP risk, we determined whether intervention effects varied by demographic characteristics, particularly those identified as covariates. Overall, the results failed to identify effects as a moderating function of age (*P*=.31), sex (*P*=.35), having a child (*P*=.90), basic expenses (*P*=.26), education level (*P*=.06), numeracy level (*P*=.06), or current tobacco use (*P*=.41). A similar analysis for perceived NETP risk indicated no effects as a function of age (*P*=.51), sex (*P*=.64), having a child (*P*=.75), basic expenses (*P*=.14), education level (*P*=.87), numeracy level (*P*=.05), or current tobacco use (*P*=.26).

### Change in Perceived NETP Risk by Type of Message Received

As presented in Table 2, there was a significant increase in perceived NETP risk among participants receiving emotional messages regarding NETPs (*P*=.006). We also observed a nonsignificant increase in perceived NETP risk among participants who received complex messages (*P*=.01) and those who received loss-framed messages (*P*=.01).

Table 3 shows a significant increase in perceived NETP risk among participants who received rational messages (*P*=.005), simple messages (*P*=.003), and gain-framed messages regarding NETPs (*P*=.003).

**Table 2:** Change in perceived risk of using NETP^a^ among participants receiving emotional messages, those receiving complex messages, and those receiving loss-framed messages.^b^

Characteristics			Emotional (n=314)				Complex (n=324)				Loss-framed (n=321)		
			B (SE)		*P* value		B (SE)		*P* value		B (SE)		*P* value
Time^c^			0.24 (0.09)		.006		0.21 (0.08)		.01		0.20 (0.08)		.01
Crossover group			0.03 (0.09)		.72		−0.09 (0.09)		.32		−0.04 (0.09)		.63
Crossover group by time			−0.11 (0.13)		.41		0.11 (0.12)		.34		0.19 (0.12)		.11
Age			0.02 (0.02)		.23		0.02 (0.02)		.15		0.02 (0.02)		.18
Being female			−0.12 (0.08)		.14		0.04 (0.08)		.61		0.08 (0.08)		.29
Having a child			0.04 (0.13)		.76		−0.22 (0.13)		.09		0.16 (0.13)		.23
**Basic expenses**													
	Just meet	0.06 (0.15)		.66		0.06 (0.17)		.71		0.09 (0.14)		.54	
	Meet adequately	0.08 (0.15)		.59		0.11 (0.17)		.51		0.04 (0.14)		.78	
	Meet comfortably	0.09 (0.16)		.55		0.04 (0.17)		.84		0.05 (0.15)		.72	
	Cannot meet (reference)	—^d^		—		—		—		—		—	
**Education plan**													
	Associate degree	−0.25 (0.21)		.23		−0.04 (0.20)		.84		−0.29 (0.21)		.17	
	Bachelor’s degree	−0.26 (0.19)		.17		−0.22 (0.18)		.22		−0.22 (0.19)		.24	
	Master’s degree	−0.23 (0.19)		.20		−0.22 (0.18)		.22		−0.26 (0.19)		.15	
	Doctorate degree	−0.23 (0.19)		.22		−0.19 (0.18)		.28		−0.25 (0.19)		.18	
	Certificate (reference)	—		—		—		—		—		—	
Numeracy level			0.03 (0.02)		.15		−0.02 (0.02)		.36		−0.02 (0.02)		.43
Baseline NETP use^e^			−0.22 (0.09)		.009		−0.16 (0.08)		.06		−0.02 (0.08)		.01
Receive simple messages^f^			0.06 (0.07)		.41		—		—		0.15 (0.07)		.04
Receive gain-framed messages^f^			0.16 (0.07)		.04		0.13 (0.07)		.08		—		—
Receive emotional messages^f^			—		—		0.09 (0.07)		.20		−0.02 (0.07)		.78

^a^NETP: new and emerging tobacco product.

^b^Three models are presented in this table. Unstandardized coefficients are presented with the significance level at .007. Multimedia Appendix 5 presents 95% CIs for each coefficient.

^c^The unadjusted time effects for participants receiving emotional, complex, and loss-framed messages were B(SE)=0.18 (0.06), *P*=.004, B(SE)=0.26 (0.06), *P*<.001, and B(SE)=0.30 (0.06), *P*<.001, respectively.

^d^For reference factors (eg, cannot meet), this indicates that data is not applicable. For actual variables (eg, receive simple messages), this indicates that the variable was not included in the model.

^e^Baseline NETP use indicates past 30-day use of NETPs at baseline.

^f^These variables compare receiving 1 message type with its counterpart (gain-framed vs loss-framed, emotional vs rational, and simple vs complex).

**Table 3:** Change in perceived risk of using NETP^a^ among participants receiving rational messages, those receiving simple messages, and those receiving gain-framed messages.^b^

Characteristics			Rational (n=322)					Simple (n=312)					Gain-framed (n=315)		
			B (SE)		*P* value		B (SE)			*P* value		B (SE)			*P* value
Time^c^			0.22 (0.08)		.005		0.25 (0.08)			.003		0.25 (0.08)			.002
Crossover group			−0.12 (0.09)		.16		−0.02 (0.09)			.85		−0.04 (0.09)			.65
Crossover group by time			0.14 (0.11)		.21		−0.08 (0.12)			.50		−0.14 (0.12)			.24
Age			0.02 (0.02)		.16		0.02 (0.02)			.27		0.02 (0.02)			.17
Being female			0.07 (0.08)		.36		−0.07 (0.08)			.39		−0.10 (0.08)			.21
Having a child			−0.16 (0.13)		.22		0.12 (0.13)			.33		−0.22 (0.13)			.10
**Basic expenses**															
	Just meet	0.01 (0.15)		.52		0.10 (0.14)			.45		0.04 (0.16)			.80	
	Meet adequately	0.16 (0.15)		.28		0.15 (0.14)			.28		0.20 (0.16)			.22	
	Meet comfortably	0.09 (0.16)		.58		0.16 (0.14)			.27		0.13 (0.16)			.43	
	Cannot meet (reference)	—^d^		—		—			—		—			—	
**Education plan**															
	Associate degree	0.04 (0.20)		.82		−0.17 (0.20)			.39		0.07 (0.19)			.71	
	Bachelor’s degree	−0.02 (0.18)		.88		−0.04 (0.18)			.83		−0.09 (0.17)			.60	
	Master’s degree	−0.001 (0.18)		.99		0.03 (0.18)			.87		−0.01 (0.17)			.96	
	Doctorate degree	0.04 (0.18)		.80		0.03 (0.17)			.87		0.04 (0.17)			.82	
	Certificate (reference)	—		—		—			—		—			—	
Numeracy level			−0.003 (0.02)		.86		0.04 (0.02)			.04		0.03 (0.02)			.13
Baseline NETP use^e^			−0.09 (0.09)		.31		−0.16 (0.09)			.06		−0.10 (0.09)			.26
Receive simple messages^f^			0.21 (0.07)		.003		—			—		0.07 (0.07)			.30
Receive gain-framed messages^f^			0.07 (0.07)		.32		0.09 (0.07)			.04		—			—
Receive emotional messages^f^			—		—		−0.07 (0.07)			.34		0.03 (0.07)			.67

^a^NETP: new and emerging tobacco product.

^b^Three models are presented in this table. Unstandardized coefficients are presented, and the significance level is examined at .007. Multimedia Appendix 5 presents 95% CIs for each coefficient.

^c^The unadjusted time effects among participants receiving rational, simple, and gain-framed messages were B(SE)=0.30 (0.06), *P*<.001, B(SE)=0.22 (0.06), *P*<.001, and B (SE)=0.18 (0.06), *P*=.002, respectively.

^d^For reference factors (eg, cannot meet), this indicates that data is not applicable. For actual variables (eg, receive simple messages), this indicates that the variable was not included in the model.

^e^Baseline NETP use indicates past 30-day use of NETPs at baseline.

^f^These variables compare receiving 1 message type with its counterpart (gain-framed vs loss-framed, emotional vs rational, and simple vs complex).

### Change in Perceived CTP Risk by Type of Message Received

Table 4 presents an observable nonsignificant increase in perceived CTP risk among participants who received emotional messages (*P*=.01), those who received complex messages (*P*=.03), and those who received loss-framed messages (*P*=.01). Among participants receiving complex messages, higher numeracy levels were significantly related to an increase in perceived CTP risk (*P*=.006).

On the other hand, as presented in Table 5, there was no significant increase in perceived CTP risk among participants who received rational (*P*=.20), simple (*P*=.11), or gain-framed messages (*P*=.23). Among participants who received simple messages, numeracy level (*P*=.006) and current CTP use (*P*<.001) were significantly related to higher perceived CTP risk.

**Table 4:** Change in perceived risk of using CTP^a^ among participants receiving emotional messages, those receiving complex messages, and those receiving loss-framed messages.^b^

Characteristics			Emotional (n=314)				Complex (n=324)				Loss-framed (n=321)		
			B (SE)		*P* value		B (SE)		*P* value		B (SE)		*P* value
Time^c^			0.02 (0.07)		.01		0.16 (0.07)		.03		0.18 (0.07)		.01
Crossover group			0.17 (0.07)		.76		−0.09 (0.08)		.24		−0.01 (0.08)		.94
Crossover group by time			−0.05 (0.10)		.64		0.03 (0.10)		.76		0.06 (0.10)		.54
Age			−0.01 (0.01)		.36		−0.01 (0.02)		.66		−0.01 (0.02)		.63
Being female			−0.01 (0.07)		.94		0.05 (0.07)		.45		0.04 (0.07)		.54
Having a child			0.11 (0.11)		.33		0.04 (0.12)		.71		0.19 (0.11)		.09
**Basic expenses**													
	Just meet	0.25 (0.13)		.06		0.12 (0.15)		.43		0.22 (0.13)		.11	
	Meet adequately	0.27 (0.13)		.04		0.24 (0.15)		.11		0.23 (0.13)		.07	
	Meet comfortably	0.13 (0.14)		.35		0.11 (0.15)		.47		0.08 (0.13)		.55	
	Cannot meet (reference)	—^d^		—		—		—		—		—	
**Education plan**													
	Associate degree	−0.08 (0.18)		.68		0.19 (0.17)		.27		0.04 (0.18)		.80	
	Bachelor’s degree	−0.002 (0.16)		.99		0.06 (0.16)		.71		−0.002 (0.16)		.99	
	Master’s degree	0.06 (0.16)		.71		0.08 (0.15)		.59		0.01 (0.16)		.96	
	Doctorate degree	−0.04 (0.16)		.79		0.10 (0.16)		.52		−0.04 (0.16)		.81	
	Certificate (reference)	—		—		—		—		—		—	
Numeracy level			0.02 (0.02)		.28		−0.02 (0.02)		.31		0.01 (0.02)		.52
Baseline CTP use^e^			−0.16 (0.09)		.08		0.07 (0.09)		.45		−0.14 (0.09)		.13
Receive simple messages^f^			0.04 (0.06)		.53		—		—		0.06 (0.06)		.47
Receive gain-framed messages^f^			0.14 (0.07)		.04		0.04 (0.07)		.57		—		—
Receive emotional messages^f^			—		—		0.06 (0.07)		.32		−0.05 (0.06)		.47

^a^CTP: conventional tobacco product.

^b^Three models are presented in this table. Unstandardized coefficients are presented, and the significance level is examined at .007. Multimedia Appendix 5 presents 95% CIs for each coefficient.

^c^The unadjusted time effects among participants receiving emotional, complex, and loss-framed messages were B (SE)=0.16 (0.05), *P*=.001, B (SE)=0.17 (0.05), *P*=.001, and B (SE)=0.21 (0.05), *P*<.001, respectively.

^d^For reference factors (eg, cannot meet), this indicates that data is not applicable. For actual variables (eg, receive simple messages), this indicates that the variable was not included in the model.

^e^Baseline CTP use indicates past 30-day use of CTPs at baseline.

^f^These variables compare receiving 1 message type with its counterpart (gain-framed vs loss-framed, emotional vs rational, and simple vs complex).

**Table 5:** Change in perceived risk of using CTP^a^ among participants receiving rational messages, those receiving simple messages, and those receiving gain-framed messages.^b^

Characteristics		Rational (n=322)			Simple (n=312)			Gain-framed (n=315)	
		B (SE)	*P* value	B (SE)		*P* value	B (SE)		*P* value
Time^c^		0.09 (0.07)	.20	0.11 (0.07)		.12	0.08 (0.07)		.23
Crossover group		−0.10 (0.07)	.19	0.02 (0.07)		.81	−0.06 (0.07)		.41
Crossover group by time		0.12 (0.10)	.22	0.05 (0.10)		.62	0.03 (0.10)		.75
Age		0.01 (0.01)	.31	0.01 (0.01)		.48	0.01 (0.01)		.65
Being female		0.04 (0.06)	.49	−0.02 (0.06)		.81	−0.002 (0.07)		.98
Having a child		0.08 (0.11)	.47	0.18 (0.10)		.07	0.02 (0.11)		.84
**Basic expenses**									
	Just meet	−0.14 (0.12)	.25	0.06 (0.11)		.58	−0.12 (0.13)		.36
	Meet adequately	0.01 (0.13)	.96	0.10 (0.11)		.36	0.04 (0.13)		.78
	Meet comfortably	−0.13 (0.13)	.31	−0.05 (0.11)		.67	−0.11 (0.14)		.42
	Cannot meet (reference)	—^d^	—	—		—	—		—
**Education plan**									
	Associate degree	0.31 (0.16)	.05	−0.001 (0.16)		1.00	0.18 (0.16)		.26
	Bachelor’s degree	0.28 (0.14)	.05	0.24 (0.14)		.09	0.24 (0.14)		.09
	Master’s degree	0.29 (0.14)	.04	0.32 (0.14)		.02	0.32 (0.14)		.02
	Doctorate degree	0.35 (0.14)	.01	0.24 (0.14)		.08	0.32 (0.14)		.02
	Certificate (reference)	—	—	—		—	—		—
Numeracy level		0.01 (0.02)	.65	0.04 (0.01)		.004	0.01 (0.02)		.37
Baseline CTP use^e^		−0.03 (0.08)	.74	−0.30 (0.08)		<.001	−0.05 (0.08)		.51
Receive simple messages^f^		0.09 (0.06)	.11	—		—	0.06 (0.06)		.36
Receive gain-framed messages^f^		−0.03 (0.06)	.64	0.10 (0.06)		.09	—		—
Receive emotional messages^f^		—	—	−0.01 (0.06)		.89	0.09 (0.06)		.37

^a^CTP: conventional tobacco product.

^b^Three models are presented in this table. Unstandardized coefficients are presented with the significance level at .007. Multimedia Appendix 5 presents 95% CIs for each coefficient.

^c^The unadjusted time effects among participants receiving rational, simple, and gain-framed messages were B(SE)=0.16 (0.05), *P*=.002, B(SE)=0.14 (0.05), *P*=.004, and B (SE)=0.10 (0.05), *P*=.03, respectively.

^d^For reference factors (eg, cannot meet), this indicates that data is not applicable. For actual variables (eg, receive simple messages), this indicates that the variable was not included in the model.

^e^Baseline CTP use indicates past 30-day use of CTPs at baseline.

^f^These variables compare receiving 1 message type with its counterpart (gain-framed vs loss-framed, emotional vs rational, and simple vs complex).

## Discussion

### Principal Findings

Tobacco marketing has successfully crafted messages to promote tobacco use among the general public, particularly among YAs. As a result, there has been evidence of limited public knowledge concerning the harms of tobacco products [82,83], particularly NETPs such as e-cigarettes and hookahs [84]. The use of mobile phones in the United States is nearly ubiquitous. This study expands on previous research by identifying exceptionally successful types of SMS text messages that can be used to correct YAs’ perceptions of tobacco risk. Controlling for the type of message received, our results show that YAs had a significant increase in perceived NETP risk. However, when stratifying by message type, we observed a significant increase over time in perceived NETP risk upon exposure to emotional, rational, simple, and gain-framed messages. In addition, YAs generally had an increase in perceived CTP risk. Although not significant, after stratification, we observed an increase in perceived CTP risk upon exposure to emotional, complex, and loss-framed messages.

Previous research on risk perception among YAs supports our results pertaining to emotional and rational messages [85], and it is in line with the ELM of persuasion [61]. In particular, the harms of NETPs are still unfamiliar to young populations and YAs. To avoid a higher-level effort to process messages regarding NETPs [86], participants receiving simple messages had an increase in perceived NETP risk, whereas participants receiving complex messages did not. This supports the need for simple messages to convey information that requires more effort to understand. Similarly, with a need to engage in peripheral processing, participants who received emotional messages also improved in perceived NETP risk. Interestingly, rational messages also produced a significant increase in perceived NETP risk, indicating the success of central processing of information on NETPs. Conversely, YAs tend to be more familiar with the harms of CTPs [86]. With less need for effortful cognitive information processing, although nonsignificant, participants receiving emotional messages and those receiving complex messages produced an observable increase in perceived CTP risk, whereas participants receiving rational messages and those receiving simple messages did not. Although the ELM may explain these results, it is key for future researchers to further examine this interpretation and measure YAs’ need for cognition and familiarity with the products.

However, according to previous research, both gain-framed and loss-framed messages can be effective in increasing tobacco risk perception through different mechanisms [87,88]. In this study, although not significant, our results indicate an observable decrease in perceived CTP risk among YAs exposed to loss-framed messages. Supportive of our findings, previous research has frequently indicated that YAs favor loss-framed messages with health risk themes [88,89]. By highlighting the potential losses that result from tobacco use, it is expected that YAs experience sadness and fear, thereby increasing risk perception [87]. On the other hand, YAs who received gain-framed messages had significantly improved perceived NETP risk levels. This finding agrees with previous research indicating that emphasis on the potential benefits of avoiding tobacco can stimulate a sense of guilt responsible for a decrease in risk perception [83]. In addition, the success of our gain-framed messages can be attributed to their design. In particular, these messages did not directly present the benefits gained as a result of avoiding tobacco (ie, not using tobacco helps one gain certain benefits). Instead, most messages described a lack of loss as being the benefit (ie, not using tobacco helps one avoid negative consequences). This type of message framing takes advantage of a gain-framed design while still describing losses. Regardless of the mechanism at play, our results emphasize the effectiveness of messages that communicate a need to *avoid losses* resulting from NETP use. Future research should consider implementing this message structure to improve the perceived NETP risk.

### Limitations

There are some study limitations to be considered. First, this study involved a convenience sample. Nevertheless, the sample is representative of the diverse community college population in terms of demographic characteristics and tobacco use among Texan YAs [90,91]. Second, the trial included a wide variety of tobacco products, making it difficult to attribute the outcomes to messages on specific products. However, the distinction between messages on NETPs (eg, vaping products and hookah) and CTPs (combustible and smokeless products) made it possible to identify successful message types for these 2 common groups of products in the United States. Finally, the loss of participants to follow-up with retention of only 70.1% (446/636) of participants may have made it difficult to capture the significance of some of the observed predictions.

### Implications

With our current findings, we cannot conclude that one message type is more effective than another. Nevertheless, this study aimed to identify successful message types individually. Our results suggest that specific types of SMS text messages can be particularly successful. On the basis of our findings, we encourage future researchers to apply emotional, complex, and loss-framed messages when conveying the harm of CTPs. On the other hand, we recommend the use of simple and gain-framed messages to inform about the harms of NETPs. These messages may be emotional or rational.

Our messages can be strategically disseminated within campaigns conducted via social media, smartphone apps, or mass media. Our previous research has posited that YAs are interested in mobile health (mHealth) programs that help them learn about tobacco risks [58], and mHealth programs offer the potential to greatly increase the reach of YAs. Such mHealth programming can present rational, simple, and gain-framed messages for communication of the risk of NETPs. Conversely, emotional, complex, and loss-framed messages can be disseminated to communicate the risk of CTPs.

It is important to note that the appropriateness and impact of the messages are likely to be context-dependent, and the results may have limited transferability. Nevertheless, with the results of this study, researchers and practitioners implementing mHealth programs may take advantage of our tailored messages through larger technology-based programs such as smartphone apps and social media campaigns. If a program were to be designed where individuals could opt in to receive the messages, a separate study might be needed to examine the target populations’ needs and preferences with respect to these messages. One promising avenue for future research in this area is the integration of these messages into narratives that can facilitate accurate tobacco risk perception. Several studies have begun to consider the investigation of message framing strategies within narratives, indicating that narratives can be successful with both loss-framed and gain-framed messages [88]. In the next step, we plan to examine how the success of narratives can be improved based on message complexity and emotional appeal. Although mHealth SMS text messaging can efficiently and widely communicate tobacco risk, by integrating narrative-based messages, researchers are likely to improve YAs’ engagement through message attention and recall of information.

## Appendix

Multimedia Appendix 1 Description and examples of SMS text messages.

Multimedia Appendix 2 CONSORT-eHEALTH checklist (V 1.6.1).

Multimedia Appendix 3 Associations between population characteristics and primary outcomes.

Multimedia Appendix 4 Demographic characteristics and risk perception ratings.

Multimedia Appendix 5 The 95% CIs for each coefficient and for all tables.

## Abbreviations

CONSORT: Consolidated Standards of Reporting Trials
CONSORT-EHEALTH: CONSORT–Electronic and Mobile Health Applications and Online TeleHealth
CTP: conventional tobacco product
ELM: elaboration likelihood model
mHealth: mobile health
NETP: new and emerging tobacco product
YA: young adult

## References

[1] Prokhorov AV, Khalil GE, Foster DW, Marani SK, Guindani M, Espada JP, Gonzálvez MT, Idrisov B, Galimov A, Arora M, Tewari A, Isralowitz R, Lapvongwatana P, Chansatitporn N, Chen X, Zheng H, Sussman S (2017). Testing the nicotine dependence measure mFTQ for adolescent smokers: a multinational investigation. Am J Addict.

[2] Dierker L, Rose J, Selya A, Piasecki TM, Hedeker D, Mermelstein R (2015). Depression and nicotine dependence from adolescence to young adulthood. Addict Behav.

[3] Viswam D, Trotter S, Burge PS, Walters GI (2018). Respiratory failure caused by lipoid pneumonia from vaping e-cigarettes. BMJ Case Rep.

[4] King BA, Jones CM, Baldwin GT, Briss PA (2020). The EVALI and youth vaping epidemics - implications for public health. N Engl J Med.

[5] Klakk H, Kristensen PL, Andersen LB, Froberg K, Møller NC, Grøntved A (2018). Symptoms of depression in young adulthood is associated with unfavorable clinical- and behavioral cardiovascular disease risk factors. Prev Med Rep.

[6] Carlos WG, Crotty Alexander LE, Gross JE, Dela Cruz CS, Keller JM, Pasnick S, Jamil S (2019). ATS Health Alert-Vaping-associated Pulmonary Illness (VAPI). Am J Respir Crit Care Med.

[7] Hooper RW, Garfield JL (2020). An emerging crisis: vaping-associated pulmonary injury. Ann Intern Med.

[8] Leventhal AM, Strong DR, Kirkpatrick MG, Unger JB, Sussman S, Riggs NR, Stone MD, Khoddam R, Samet JM, Audrain-McGovern J (2015). Association of electronic cigarette use with initiation of combustible tobacco product smoking in early adolescence. JAMA.

[9] Leventhal AM, Stone MD, Andrabi N, Barrington-Trimis J, Strong DR, Sussman S, Audrain-McGovern J (2016). Association of e-cigarette vaping and progression to heavier patterns of cigarette smoking. JAMA.

[10] Goldenson NI, Leventhal AM, Stone MD, McConnell RS, Barrington-Trimis JL (2017). Associations of electronic cigarette nicotine concentration with subsequent cigarette smoking and vaping levels in adolescents. JAMA Pediatr.

[11] Soneji S, Barrington-Trimis JL, Wills TA, Leventhal AM, Unger JB, Gibson LA, Yang J, Primack BA, Andrews JA, Miech RA, Spindle TR, Dick DM, Eissenberg T, Hornik RC, Dang R, Sargent JD (2017). Association between initial use of e-cigarettes and subsequent cigarette smoking among adolescents and young adults: a systematic review and meta-analysis. JAMA Pediatr.

[12] Schulenberg J, Johnston L, O'Malley P, Bachman J, Miech R, Patrick M (2020). Monitoring the Future National Survey Results on Drug Use, 1975-2019: Volume II, College Students and Adults Ages 19-60.

[13] Berg CJ, Stratton E, Schauer GL, Lewis M, Wang Y, Windle M, Kegler M (2015). Perceived harm, addictiveness, and social acceptability of tobacco products and marijuana among young adults: marijuana, hookah, and electronic cigarettes win. Subst Use Misuse.

[14] Tan AS, Mello S, Sanders-Jackson A, Bigman CA (2017). Knowledge about chemicals in e-cigarette secondhand vapor and perceived harms of exposure among a national sample of U.S. Adults. Risk Anal.

[15] Gowin M, Cheney MK, Wann TF (2017). Knowledge and beliefs about e-cigarettes in straight-to-work young adults. Nicotine Tob Res.

[16] Pokhrel P, Lam TH, Pagano I, Kawamoto CT, Herzog TA (2018). Young adult e-cigarette use outcome expectancies: validity of a revised scale and a short scale. Addict Behav.

[17] Hair E, Rath JM, Pitzer L, Emelle B, Ganz O, Halenar MJ, Cantrell J, Vallone D (2017). Trajectories of hookah use: harm perceptions from youth to young adulthood. Am J Health Behav.

[18] O'Brien EK, Nguyen AB, Persoskie A, Hoffman AC (2017). U.S. adults' addiction and harm beliefs about nicotine and low nicotine cigarettes. Prev Med.

[19] McDonald EA, Ling PM (2015). One of several 'toys' for smoking: young adult experiences with electronic cigarettes in New York City. Tob Control.

[20] Rodu B, Plurphanswat N, Hughes JR, Fagerström K (2016). Associations of proposed relative-risk warning labels for snus with perceptions and behavioral intentions among tobacco users and nonusers. Nicotine Tob Res.

[21] Wagoner KG, Cornacchione J, Wiseman KD, Teal R, Moracco KE, Sutfin EL (2016). E-cigarettes, hookah pens and vapes: adolescent and young adult perceptions of electronic nicotine delivery systems. Nicotine Tob Res.

[22] Berry C, Burton S, Howlett E (2017). Are cigarette smokers', e-cigarette users', and dual users' health-risk beliefs and responses to advertising influenced by addiction warnings and product type?. Nicotine Tob Res.

[23] Kozlowski LT, Sweanor DT (2018). Young or adult users of multiple tobacco/nicotine products urgently need to be informed of meaningful differences in product risks. Addict Behav.

[24] Wong EC, Haardörfer R, Windle M, Berg CJ (2017). Distinct motives for use among polytobacco versus cigarette only users and among single tobacco product users. Nicotine Tob Res.

[25] Noland M, Ickes MJ, Rayens MK, Butler K, Wiggins AT, Hahn EJ (2016). Social influences on use of cigarettes, e-cigarettes, and hookah by college students. J Am Coll Health.

[26] Mays D, Arrazola RA, Tworek C, Rolle IV, Neff LJ, Portnoy DB (2016). Openness to using non-cigarette tobacco products among U.S. young adults. Am J Prev Med.

[27] Loukas A, Paddock EM, Li X, Harrell MB, Pasch KE, Perry CL (2019). Electronic nicotine delivery systems marketing and initiation among youth and young adults. Pediatrics.

[28] Pierce JP, Sargent JD, Portnoy DB, White M, Noble M, Kealey S, Borek N, Carusi C, Choi K, Green VR, Kaufman AR, Leas E, Lewis MJ, Margolis KA, Messer K, Shi Y, Silveira ML, Snyder K, Stanton CA, Tanski SE, Bansal-Travers M, Trinidad D, Hyland A (2018). Association between receptivity to tobacco advertising and progression to tobacco use in youth and young adults in the path study. JAMA Pediatr.

[29] O'Brien EK, Hoffman L, Navarro MA, Ganz O (2020). Social media use by leading US e-cigarette, cigarette, smokeless tobacco, cigar and hookah brands. Tob Control.

[30] Padon AA, Maloney EK, Cappella JN (2017). Youth-targeted e-cigarette marketing in the US. Tob Regul Sci.

[31] Bahreinifar S, Sheon NM, Ling PM (2013). Is snus the same as dip? Smokers' perceptions of new smokeless tobacco advertising. Tob Control.

[32] Carter OB, Donovan R, Jalleh G (2011). Using viral e-mails to distribute tobacco control advertisements: an experimental investigation. J Health Commun.

[33] Perez DA, Grunseit AC, Rissel C, Kite J, Cotter T, Dunlop S, Bauman A (2012). Tobacco promotion 'below-the-line': exposure among adolescents and young adults in NSW, Australia. BMC Public Health.

[34] Jane LM, Bover MM, Delnevo CD (2015). Tobacco industry direct mail receipt and coupon use among young adult smokers. Prev Med.

[35] Richardson A, Ganz O, Pearson J, Celcis N, Vallone D, Villanti AC (2015). How the industry is marketing menthol cigarettes: the audience, the message and the medium. Tob Control.

[36] Richardson A, Ganz O, Vallone D (2015). Tobacco on the web: surveillance and characterisation of online tobacco and e-cigarette advertising. Tob Control.

[37] Carpenter CM, Wayne GF, Pauly JL, Koh HK, Connolly GN (2005). New cigarette brands with flavors that appeal to youth: tobacco marketing strategies. Health Aff (Millwood).

[38] Ling PM, Glantz SA (2002). Why and how the tobacco industry sells cigarettes to young adults: evidence from industry documents. Am J Public Health.

[39] Rainie L, Perrin A (2017). 10 facts about smartphones as the iPhone turns 10. Pew Research Center.

[40] Reynolds R Camel Snus: we had a feeling you would be stopping. https://snus.tobaccopleasure.com/modules/.

[41] Marketing Practices. Philip Morris USA.

[42] Sears CG, Walker KL, Hart JL, Lee AS, Siu A, Smith C (2017). Clean, cheap, convenient: promotion of electronic cigarettes on YouTube. Tob Prev Cessat.

[43] Huang J, Kornfield R, Emery SL (2016). 100 Million Views of Electronic Cigarette YouTube Videos and Counting: Quantification, Content Evaluation, and Engagement Levels of Videos. J Med Internet Res.

[44] BinDhim NF, Freeman B, Trevena L (2015). Pro-smoking apps: where, how and who are most at risk. Tob Control.

[45] Research priorities. US Food and Drug Administration.

[46] Ashley DL, Backinger CL, van Bemmel DM, Neveleff DJ (2014). Tobacco regulatory science: research to inform regulatory action at the Food and Drug Administration's Center for Tobacco Products. Nicotine Tob Res.

[47] Muench F, Weiss RA, Kuerbis A, Morgenstern J (2013). Developing a theory driven text messaging intervention for addiction care with user driven content. Psychol Addict Behav.

[48] Scott-Sheldon LAJ, Lantini R, Jennings EG, Thind H, Rosen RK, Salmoirago-Blotcher E, Bock BC (2016). Text messaging-based interventions for smoking cessation: a systematic review and meta-analysis. JMIR Mhealth Uhealth.

[49] Obermayer JL, Riley WT, Asif O, Jean-Mary J (2004). College smoking-cessation using cell phone text messaging. J Am Coll Health.

[50] Riley W, Obermayer J, Jean-Mary J (2008). Internet and mobile phone text messaging intervention for college smokers. J Am Coll Health.

[51] Sandrick J, Tracy D, Eliasson A, Roth A, Bartel J, Simko M, Bowman T, Harouse-Bell K, Kashani M, Vernalis M (2017). Effect of a counseling session bolstered by text messaging on self-selected health behaviors in college students: a preliminary randomized controlled trial. JMIR Mhealth Uhealth.

[52] Head KJ, Noar SM, Iannarino NT, Grant HN (2013). Efficacy of text messaging-based interventions for health promotion: a meta-analysis. Soc Sci Med.

[53] Armanasco AA, Miller YD, Fjeldsoe BS, Marshall AL (2017). Preventive health behavior change text message interventions: a meta-analysis. Am J Prev Med.

[54] Latimer AE, Krishnan-Sarin S, Cavallo DA, Duhig A, Salovey P, O'Malley SA (2012). Targeted smoking cessation messages for adolescents. J Adolesc Health.

[55] Toll BA, Salovey P, O'Malley SS, Mazure CM, Latimer A, McKee SA (2008). Message framing for smoking cessation: the interaction of risk perceptions and gender. Nicotine Tob Res.

[56] Biener L, McCallum-Keeler G, Nyman AL (2000). Adults' response to Massachusetts anti-tobacco television advertisements: impact of viewer and advertisement characteristics. Tob Control.

[57] Steward WT, Schneider TR, Pizarro J, Salovey P (2003). Need for cognition moderates responses to framed smoking-cessation messages. J Appl Social Pyschol.

[58] Prokhorov AV, Machado TC, Calabro KS, Vanderwater EA, Vidrine DJ, Pasch KP, Marani SK, Buchberg M, Wagh A, Russell SC, Czerniak KW, Botello GC, Dobbins MH, Khalil GE, Perry CL (2017). Developing mobile phone text messages for tobacco risk communication among college students: a mixed methods study. BMC Public Health.

[59] Khalil GE, Calabro KS, Crook B, Machado TC, Perry CL, Prokhorov AV (2018). Validation of mobile phone text messages for nicotine and tobacco risk communication among college students: a content analysis. Tob Prev Cessat.

[60] Prokhorov AV, Khalil GE, Calabro KS, Machado TC, Russell S, Czerniak KW, Botello GC, Chen M, Perez A, Vidrine DJ, Perry CL (2018). Mobile phone text messaging for tobacco risk communication among young adult community college students: protocol and baseline overview for a randomized controlled trial. JMIR Res Protoc.

[61] Petty R, Cacioppo J (1986). The elaboration likelihood model of persuasion. Advances in Experimental Social Psychology.

[62] O'Keefe D (2008). Elaboration likelihood model. The International Encyclopedia of Communication.

[63] Chen S-, Lee K- (2008). The role of personality traits and perceived values in persuasion: an elaboration likelihood model perspective on online shopping. Soc Behav Personal.

[64] Lang A, Yegiyan NS (2008). Understanding the interactive effects of emotional appeal and claim strength in health messages. J Broadcasting Elect Media.

[65] Kahneman D, Tversky A, MacLean LC, Ziemba WT (2013). Prospect theory: an analysis of decision under risk. Handbook of The Fundamentals of Financial Decision Making.

[66] Kühberger (1998). The influence of framing on risky decisions: a meta-analysis. Organ Behav Hum Decis Process.

[67] O'Keefe DJ, Jensen JD (2007). The relative persuasiveness of gain-framed and loss-framed messages for encouraging disease prevention behaviors: a meta-analytic review. J Health Commun.

[68] Gallagher KM, Updegraff JA (2012). Health message framing effects on attitudes, intentions, and behavior: a meta-analytic review. Ann Behav Med.

[69] Eysenbach G (2013). CONSORT-EHEALTH: implementation of a checklist for authors and editors to improve reporting of web-based and mobile randomized controlled trials. Stud Health Technol Inform.

[70] Eysenbach G, CONSORT-EHEALTH Group (2011). CONSORT-EHEALTH: improving and standardizing evaluation reports of Web-based and mobile health interventions. J Med Internet Res.

[71] (2017). Houston community college fact book. Houston Community College.

[72] Johnston LD, Malley PM, Bachman JG, Schulenberg JE, Miech RA (2016). Monitoring the future national survey results on drug use, 1975-2015: Volume ii, college students and adults ages 19-55. Monitoring the Future.

[73] Nelson W, Reyna VF, Fagerlin A, Lipkus I, Peters E (2008). Clinical implications of numeracy: theory and practice. Ann Behav Med.

[74] Wray RJ, Jupka K, Berman S, Zellin S, Vijaykumar S (2012). Young adults' perceptions about established and emerging tobacco products: results from eight focus groups. Nicotine Tob Res.

[75] Rath JM, Villanti AC, Abrams DB, Vallone DM (2012). Patterns of tobacco use and dual use in US young adults: the missing link between youth prevention and adult cessation. J Environ Public Health.

[76] Johnston L, O'Malley P, Bachman J, Schulenberg J (2009). Monitoring the Future National Results on Adolescent Drug Use: Overview of Key Findings.

[77] Loukas A, Batanova M, Fernandez A, Agarwal D (2015). Changes in use of cigarettes and non-cigarette alternative products among college students. Addict Behav.

[78] Kutner MH, Nachtsheim CJ, Neter J, Li W (2005). Applied linear statistical models.

[79] Groenwold RH, Goeman JJ, Cessie SL, Dekkers OM (2021). Multiple testing: when is many too much?. Eur J Endocrinol.

[80] Motulsky H (2010). Intuitive Biostatistics: A Nonmathematical Guide to Statistical Thinking.

[81] Feise RJ (2002). Do multiple outcome measures require p-value adjustment?. BMC Med Res Methodol.

[82] Case K, Crook B, Lazard A, Mackert M (2016). Formative research to identify perceptions of e-cigarettes in college students: implications for future health communication campaigns. J Am Coll Health.

[83] Cornacchione J, Wagoner KG, Wiseman KD, Kelley D, Noar SM, Smith MH, Sutfin EL (2016). Adolescent and young adult perceptions of hookah and little cigars/cigarillos: implications for risk messages. J Health Commun.

[84] Wiseman KD, Cornacchione J, Wagoner KG, Noar SM, Moracco KE, Teal R, Wolfson M, Sutfin EL (2016). Adolescents' and young adults' knowledge and beliefs about constituents in novel tobacco products. Nicotine Tob Res.

[85] Vidrine JI, Simmons VN, Brandon TH (2007). Construction of smoking-relevant risk perceptions among college students: the influence of need for cognition and message content. J Appl Social Pyschol.

[86] Copeland AL, Peltier MR, Waldo K (2017). Perceived risk and benefits of e-cigarette use among college students. Addict Behav.

[87] Liu S, Yang JZ (2020). Incorporating Message Framing into Narrative Persuasion to Curb E-Cigarette Use Among College Students. Risk Anal.

[88] Liu S, Yang JZ (2020). The role of temporal distance perception in narrative vs. non-narrative persuasion related to e-cigarettes. J Health Commun.

[89] Kong G, Cavallo DA, Camenga DR, Morean ME, Krishnan-Sarin S (2016). Preference for gain- or loss-framed electronic cigarette prevention messages. Addict Behav.

[90] Perry CL, Creamer MR, Chaffee BW, Unger JB, Sutfin EL, Kong G, Shang C, Clendennen SL, Krishnan-Sarin S, Pentz MA (2020). Research on youth and young adult tobacco use, 2013-2018, from the Food and Drug Administration-national institutes of health tobacco centers of regulatory science. Nicotine Tob Res.

[91] Bandiera FC, Loukas A, Li X, Wilkinson AV, Perry CL (2017). Depressive symptoms predict current e-cigarette use among college students in Texas. Nicotine Tob Res.

