# Supplementary material for: Mobile Text Messaging for Tobacco Risk Communication Among Young Adult Community College Students: Randomized Trial of Project Debunk
Source: JMIR Mhealth Uhealth. 2021 Nov 24;9(11):e25618. doi: 10.2196/25618 (PMC8663493; doi:10.2196/25618)
Supplement: Multimedia Appendix 1 [file mhealth_v9i11e25618_app1.docx]

**Multimedia Appendix 1:**

**Examples of Text Messages Used in Our Protocol:**

|  | **Gain-framed** | | **Loss-framed** | |
| --- | --- | --- | --- | --- |
|  | **Simple** | **Complex** | **Simple** | **Complex** |
| **Emotional** | Enjoy the feel of a healthy heart by not smoking cigs! Lub dub…lub dub. See? Healthy hearts can work better when ppl stay away from tobacco! :) | Be astute! Savvy individuals who do not smoke hookah are less susceptible to contracting unsightly oral herpes since hookah mouthpieces are carriers for everyone's disgusting pathogens. :) | Jo, scared of germs, has seen & smelled a fungus-covered toenail. She likes fungus-free lungs but hookah exposes her to fungi that creep within hookah pipes. :( | Devastating news! Smoking "light" cigarettes will not protect the body from toxicity. ~ All cigarettes rip away approximately a decade from a smoker's lifespan :( |
| **Rational** | 16 million Americans can avoid 1 serious illness each year by not smoking. Staying away from cigs, even "lights", is the #1 way to stay healthy. | People who do not use hookah are less susceptible to contracting oral herpes from it because they are not sharing mouthpieces that carry pathogens at hookah lounges. | Hookah users expose their bodies to a lot of smoke since hookah creates 200x more smoke than a cig. | Why do the 60+ carcinogens in cigarettes pose a direct threat to smokers? Because smokers inhale those carcinogens, raising their chances of developing cancer. |

**Range of Content:**

Medical Consequences of tobacco use, mental consequences, physical appearance, electronic delivery system safety, nicotine dependence, secondhand smoke, thirdhand smoke, environmental hazards, chemical content, and financial consequences

**Distribution of Messages by Tobacco Products:**

With two categories of products, half of the messages was concerned with NETPs and the other half was concerned with CTPs. Within the messages for NETPs and the messages for CTPs, the number of messages for specific products depended on the available health information to convey, based on previous published research. In addition, it must be noted that some messages conveyed information about several products within a product category at the same time (e.g., discussing secondhand smoke). In general, within the messages for NETPs, 163 (33.4%) were exclusively on vaping products, 164 (33.6%) were exclusively on hookah, and 80 (16.4%) were exclusively on snus, 40 (8.2%) were exclusively about little cigars/cigarillos. The remaining messages conveyed information about several NETP products. Within the messages for CTPs, 304 (62.2%) were exclusively about regular cigarettes, 24 (4.9%) were exclusively about pipes, 40 (8.2%) were exclusively about cigars, and 56 (11.5%) were exclusively about chewing tobacco/dip/snuff. The remaining messages conveyed information about several CTP products.
