# Supplementary material for: Mobile Text Messaging for Tobacco Risk Communication Among Young Adult Community College Students: Randomized Trial of Project Debunk
Source: JMIR Mhealth Uhealth. 2021 Nov 24;9(11):e25618. doi: 10.2196/25618 (PMC8663493; doi:10.2196/25618)
Supplement: Multimedia Appendix 3 [file mhealth_v9i11e25618_app3.docx]

**Multimedia Appendix 3**

**Associations Between Population Characteristics and Perceived Risk at Baseline and Follow Up:**

The covariates were selected to be included in the final models when they exhibited an association with perceived risk with p<0.10 at least once at any time point.

Table: Regression models with Demographic Characteristics Predicting Perceived Risk of Using Each Conventional Tobacco Product at Baseline

|  | **Perceived CTP risk** | | | **Perceived risk of cigarette use** | | | **Perceived risk of cigar use** | | | **Perceived risk of pipe use** | | |
| --- | --- | --- | --- | --- | --- | --- | --- | --- | --- | --- | --- | --- |
|  | Beta | SE | P | Beta | SE | P | Beta | SE | P | Beta | SE | P |
| **Age** | -0.02 | 0.01 | 0.586 | -0.02 | 0.01 | 0.586 | -0.03 | 0.02 | 0.451 | -0.03 | 0.02 | 0.519 |
| **Being Female** | 0.00 | 0.06 | 0.923 | 0.01 | 0.06 | 0.846 | -0.01 | 0.07 | 0.855 | 0.02 | 0.07 | 0.656 |
| **Having a Child** | 0.05 | 0.09 | 0.212 | 0.05 | 0.08 | 0.105 | 0.06 | 0.12 | 0.121 | 0.04 | 0.12 | 0.278 |
|  |  |  |  |  |  |  |  |  |  |  |  |  |
| **Basic Expenses** |  |  |  |  |  |  |  |  |  |  |  |  |
| Just Meet | 0.10 | 0.13 | 0.269 | 0.03 | 0.13 | 0.679 | 0.12 | 0.16 | 0.185 | 0.07 | 0.16 | 0.427 |
| Meet Adequately | 0.13 | 0.14 | 0.150 | 0.04 | 0.13 | 0.609 | 0.09 | 0.16 | 0.305 | 0.09 | 0.16 | 0.294 |
| Meet Comfortably | 0.05 | 0.14 | 0.578 | 0.01 | 0.13 | 0.904 | 0.07 | 0.17 | 0.408 | 0.02 | 0.16 | 0.836 |
| Cannot meet (ref.) |  |  |  |  |  |  |  |  |  |  |  |  |
| **Education Plan** |  |  |  |  |  |  |  |  |  |  |  |  |
| Associate Degree | 0.01 | 0.18 | 0.889 | -0.03 | 0.17 | 0.696 | 0.04 | 0.20 | 0.570 | 0.01 | 0.21 | 0.902 |
| Bachelor’s Degree | -0.02 | 0.16 | 0.882 | -0.02 | 0.15 | 0.809 | -0.01 | 0.19 | 0.955 | -0.03 | 0.19 | 0.717 |
| Master’s Degree | 0.02 | 0.16 | 0.849 | -0.02 | 0.15 | 0.874 | 0.03 | 0.19 | 0.771 | 0.03 | 0.18 | 0.777 |
| Doctorate Degree | 0.02 | 0.16 | 0.853 | 0.03 | 0.15 | 0.774 | 0.00 | 0.19 | 0.998 | 0.01 | 0.18 | 0.891 |
| Certificate (ref.) |  |  |  |  |  |  |  |  |  |  |  |  |
| **Numeracy Level** | -0.002 | 0.02 | 0.968 | -0.02 | 0.02 | 0.564 | -0.01 | 0.02 | 0.734 | 0.01 | 0.02 | 0.737 |
| **Baseline Use of CTP** | -0.07 | 0.09 | 0.149 | **-0.10** | **0.09** | **0.032** | **-0.08** | **0.10** | **0.069** | -0.06 | 0.10 | 0.185 |

Table: Regression models with Demographic Characteristics Predicting Perceived Risk of Using Each Conventional Tobacco Product at Baseline (Continued)

|  | **Perceived risk of little cigars and cigarillos** | | | **Perceived risk of chewing tobacco** | | | **Perceived risk of dip-snuff** | | |
| --- | --- | --- | --- | --- | --- | --- | --- | --- | --- |
|  | Beta | SE | P | Beta | SE | P | Beta | SE | P |
| **Age** | 0.03 | 0.017 | 0.489 | -0.003 | 0.02 | 0.940 | -0.01 | 0.02 | 0.745 |
| **Being Female** | -0.002 | 0.077 | 0.960 | -0.01 | 0.07 | 0.895 | 0.004 | 0.07 | 0.925 |
| **Having a Child** | 0.03 | 0.13 | 0.532 | 0.02 | 0.11 | 0.562 | 0.02 | 0.11 | 0.647 |
|  |  |  |  |  |  |  |  |  |  |
| **Basic Expenses** |  |  |  |  |  |  |  |  |  |
| Just Meet | 0.09 | 0.17 | 0.336 | 0.12 | 0.16 | 0.215 | 0.09 | 0.14 | 0.309 |
| Meet Adequately | 0.09 | 0.171 | 0.315 | **0.18** | **0.16** | **0.055** | **0.15** | **0.14** | **0.069** |
| Meet Comfortably | 0.08 | 0.175 | 0.329 | 0.08 | 0.17 | 0.387 | 0.03 | 0.15 | 0.724 |
| Cannot meet (ref.) |  |  |  |  |  |  |  |  |  |
| **Education Plan** |  |  |  |  |  |  |  |  |  |
| Associate Degree | -0.02 | 0.20 | 0.722 | 0.01 | 0.20 | 0.911 | 0.01 | 0.20 | 0.886 |
| Bachelor’s Degree | -0.08 | 0.182 | 0.343 | -0.02 | 0.18 | 0.827 | 0.02 | 0.18 | 0.857 |
| Master’s Degree | -0.06 | 0.179 | 0.532 | 0.03 | 0.18 | 0.757 | 0.01 | 0.18 | 0.921 |
| Doctorate Degree | -0.04 | 0.18 | 0.652 | 0.03 | 0.18 | 0.764 | 0.02 | 0.18 | 0.881 |
| Certificate (ref.) |  |  |  |  |  |  |  |  |  |
| **Numeracy Level** | 0.02 | 0.02 | 0.708 | -0.004 | 0.02 | 0.914 | 0.02 | 0.02 | 0.626 |
| **Baseline Use of CTP** | -0.06 | 0.105 | 0.156 | -0.03 | 0.09 | 0.489 | -0.01 | 0.09 | 0.778 |

Table: Regression models with Demographic Characteristics Predicting Perceived Risk of Using Each Conventional Tobacco Product at Follow Up

|  | **Perceived CTP risk** | | | **Perceived risk of cigarette use** | | | **Perceived risk of cigar use** | | | **Perceived risk of pipe use** | | |
| --- | --- | --- | --- | --- | --- | --- | --- | --- | --- | --- | --- | --- |
|  | Beta | SE | P | Beta | SE | P | Beta | SE | P | Beta | SE | P |
| **Age** | 0.05 | 0.02 | 0.272 | 0.05 | 0.02 | 0.305 | 0.03 | 0.02 | 0.563 | 0.03 | 0.02 | 0.527 |
|  | 0.04 | 0.08 | 0.367 | 0.03 | 0.08 | 0.532 | 0.04 | 0.09 | 0.398 | **0.08** | **0.08** | **0.095** |
| **Having a Child** | -0.03 | 0.14 | 0.574 | -0.05 | 0.16 | 0.392 | 0.01 | 0.15 | 0.827 | -0.02 | 0.15 | 0.738 |
|  |  |  |  |  |  |  |  |  |  |  |  |  |
| **Basic Expenses** |  |  |  |  |  |  |  |  |  |  |  |  |
| Just Meet | -0.11 | 0.12 | 0.175 | -0.12 | 0.13 | 0.136 | -0.13 | 0.14 | 0.115 | -0.09 | 0.14 | 0.325 |
| Meet Adequately | 0.01 | 0.11 | 0.866 | 0.01 | 0.11 | 0.895 | 0.01 | 0.13 | 0.852 | 0.02 | 0.13 | 0.836 |
| Meet Comfortably | -0.08 | 0.12 | 0.253 | -0.08 | 0.13 | 0.279 | -0.09 | 0.14 | 0.226 | -0.07 | 0.15 | 0.384 |
| Cannot meet (ref.) |  |  |  |  |  |  |  |  |  |  |  |  |
| **Education Plan** |  |  |  |  |  |  |  |  |  |  |  |  |
| Associate Degree | -0.09 | 0.22 | 0.345 | -0.13 | 0.23 | 0.156 | -0.05 | 0.24 | 0.588 | -0.07 | 0.24 | 0.440 |
| Bachelor’s Degree | 0.00 | 0.18 | 0.978 | -0.04 | 0.18 | 0.728 | 0.01 | 0.20 | 0.902 | 0.01 | 0.20 | 0.922 |
| Master’s Degree | 0.02 | 0.18 | 0.878 | -0.01 | 0.18 | 0.899 | 0.02 | 0.20 | 0.840 | 0.04 | 0.20 | 0.746 |
| Doctorate Degree | -0.04 | 0.18 | 0.725 | -0.08 | 0.18 | 0.463 | -0.02 | 0.20 | 0.857 | 0.00 | 0.20 | 0.993 |
| Certificate (ref.) |  |  |  |  |  |  |  |  |  |  |  |  |
| **Numeracy Level** | 0.08 | 0.02 | 0.124 | 0.06 | 0.02 | 0.312 | **0.10** | **0.02** | **0.051** | 0.06 | 0.02 | 0.276 |
| **Baseline Use of CTP** | -0.08 | 0.11 | 0.169 | -0.06 | 0.12 | 0.263 | -0.08 | 0.12 | 0.129 | -0.05 | 0.11 | 0.376 |

Table: Regression models with Demographic Characteristics Predicting Perceived Risk of Using Each Conventional Tobacco Product at Follow Up (Continued)

|  | **Perceived risk of chewing tobacco** | | | **Perceived risk of dip-snuff** | | |
| --- | --- | --- | --- | --- | --- | --- |
|  | Beta | SE | P | Beta | SE | P |
| **Age** | 0.08 | 0.02 | 0.107 | 0.07 | 0.02 | 0.174 |
| **Being Female** | 0.003 | 0.08 | 0.955 | 0.05 | 0.08 | 0.295 |
| **Having a Child** | -0.04 | 0.15 | 0.518 | -0.06 | 0.16 | 0.330 |
|  |  |  |  |  |  |  |
| **Basic Expenses** |  |  |  |  |  |  |
| Just Meet | -0.07 | 0.14 | 0.442 | -0.11 | 0.13 | 0.173 |
| Meet Adequately | 0.01 | 0.13 | 0.876 | 0.004 | 0.12 | 0.953 |
| Meet Comfortably | -0.06 | 0.14 | 0.492 | -0.09 | 0.13 | 0.198 |
| Cannot meet (ref.) |  |  |  |  |  |  |
| **Education Plan** |  |  |  |  |  |  |
| Associate Degree | -0.08 | 0.23 | 0.361 | -0.08 | 0.24 | 0.407 |
| Bachelor’s Degree | 0.002 | 0.18 | 0.982 | 0.00 | 0.19 | 0.963 |
| Master’s Degree | 0.02 | 0.18 | 0.836 | 0.01 | 0.19 | 0.924 |
| Doctorate Degree | -0.04 | 0.18 | 0.741 | -0.05 | 0.19 | 0.663 |
| Certificate (ref.) |  |  |  |  |  |  |
| **Numeracy Level** | 0.06 | 0.02 | 0.276 | **0.10** | **0.02** | **0.062** |
| **Baseline Use of CTP** | **-0.10** | **0.12** | **0.07** | -0.06 | 0.12 | 0.282 |

Table: Regression models with Demographic Characteristics Predicting Perceived Risk of Using Each New and Emerging Tobacco Product at Baseline

|  | **Perceived NETP risk** | | | **Perceived risk of**  **e-cigarette use** | | | **Perceived risk of hookah use** | | |
| --- | --- | --- | --- | --- | --- | --- | --- | --- | --- |
|  | Beta | SE | P | Beta | SE | P | Beta | SE | P |
| **Age** | 0.04 | 0.02 | 0.313 | **0.07** | **0.02** | **0.083** | 0.04 | 0.02 | 0.382 |
| **Being Female** | -0.005 | 0.07 | 0.911 | 0.03 | 0.09 | 0.479 | -0.06 | 0.09 | 0.116 |
| **Having a Child^a^** | -0.01 | 0.11 | 0.840 | -0.05 | 0.15 | 0.251 | <0.0001 | 0.15 | 1.000 |
|  |  |  |  |  |  |  |  |  |  |
| **Basic Expenses** |  |  |  |  |  |  |  |  |  |
| Just Meet | 0.12 | 0.14 | 0.163 | 0.12 | 0.18 | 0.143 | 0.11 | 0.16 | 0.129 |
| Meet Adequately | 0.12 | 0.14 | 0.144 | 0.11 | 0.18 | 0.176 | 0.09 | 0.16 | 0.217 |
| Meet Comfortably | 0.10 | 0.14 | 0.208 | 0.09 | 0.18 | 0.233 | **0.12** | **0.17** | **0.084** |
| Cannot meet (ref.) |  |  |  |  |  |  |  |  |  |
| **Education Plan** |  |  |  |  |  |  |  |  |  |
| Associate Degree | -0.01 | 0.19 | 0.927 | -0.01 | 0.24 | 0.926 | -0.04 | 0.23 | 0.564 |
| Bachelor’s Degree | -0.10 | 0.18 | 0.321 | -0.11 | 0.22 | 0.227 | -0.13 | 0.21 | 0.134 |
| Master’s Degree | -0.07 | 0.18 | 0.505 | -0.09 | 0.22 | 0.357 | -0.11 | 0.21 | 0.222 |
| Doctorate Degree | -0.05 | 0.18 | 0.644 | -0.06 | 0.22 | 0.548 | -0.10 | 0.21 | 0.294 |
| Certificate (ref.) |  |  |  |  |  |  |  |  |  |
| **Numeracy Level** | -0.02 | 0.02 | 0.672 | -0.05 | 0.02 | 0.196 | -0.03 | 0.02 | 0.493 |
| **Baseline Use of NETP** | **-0.07** | **0.07** | **0.076** | **-0.08** | **0.10** | **0.057** | **-0.10** | **0.10** | **0.011** |

Note. ^a^The association between having a child and perceived NETP risk at baseline exhibited a p-value lower than 0.1 among participants who received gain-framed messages (Beta = -0.11, P = 0.054), and those who received loss-framed messages (Beta = 0.11, P = 0.057).

Table: Regression models with Demographic Characteristics Predicting Perceived Risk of Using Each New and Emerging Tobacco Product at Baseline (Continued)

|  | **Perceived risk of hookah use** | | | **Perceived risk of snus use** | | |
| --- | --- | --- | --- | --- | --- | --- |
|  | Beta | SE | P | Beta | SE | P |
| **Age** | 0.06 | 0.02 | 0.195 | -0.01 | 0.02 | 0.897 |
| **Being Female** | 0.06 | 0.09 | 0.240 | 0.02 | 0.07 | 0.558 |
| **Having a Child** | -0.02 | 0.15 | 0.666 | 0.01 | 0.11 | 0.852 |
|  |  |  |  |  |  |  |
| **Basic Expenses** |  |  |  |  |  |  |
| Just Meet | **-0.16** | **0.13** | **0.038** | 0.04 | 0.14 | 0.629 |
| Meet Adequately | -0.02 | 0.12 | 0.790 | 0.10 | 0.14 | 0.252 |
| Meet Comfortably | -0.10 | 0.13 | 0.180 | -0.003 | 0.15 | 0.971 |
| Cannot meet (ref.) |  |  |  |  |  |  |
| **Education Plan** |  |  |  |  |  |  |
| Associate Degree | -0.09 | 0.23 | 0.313 | 0.06 | 0.20 | 0.435 |
| Bachelor’s Degree | -0.04 | 0.19 | 0.674 | 0.03 | 0.19 | 0.799 |
| Master’s Degree | -0.03 | 0.19 | 0.762 | 0.06 | 0.19 | 0.609 |
| Doctorate Degree | -0.05 | 0.19 | 0.655 | 0.06 | 0.19 | 0.578 |
| Certificate (ref.) |  |  |  |  |  |  |
| **Numeracy Level** | 0.05 | 0.02 | 0.346 | 0.02 | 0.02 | 0.612 |
| **Baseline Use of NETP** | -0.02 | 0.11 | 0.668 | 0.00 | 0.07 | 0.999 |

Table: Regression models with Demographic Characteristics Predicting Perceived Risk of Using Each New and Emerging Tobacco Product at Follow Up

|  | **Perceived NETP risk** | | | **Perceived risk of**  **e-cigarette use** | | | **Perceived risk of hookah use** | | |
| --- | --- | --- | --- | --- | --- | --- | --- | --- | --- |
|  | Beta | SE | P | Beta | SE | P | Beta | SE | P |
| **Age** | **0.11** | **0.02** | **0.023** | **0.13** | **0.02** | **0.005** | **0.09** | **0.02** | **0.072** |
| **Being Female** | 0.02 | 0.08 | 0.705 | 0.01 | 0.10 | 0.789 | -0.03 | 0.10 | 0.590 |
| **Having a Child** | -0.04 | 0.15 | 0.480 | -0.03 | 0.17 | 0.601 | -0.03 | 0.18 | 0.558 |
|  |  |  |  |  |  |  |  |  |  |
| **Basic Expenses** |  |  |  |  |  |  |  |  |  |
| Just Meet | -0.06 | 0.14 | 0.490 | -0.01 | 0.19 | 0.923 | 0.02 | 0.22 | 0.880 |
| Meet Adequately | 0.01 | 0.13 | 0.886 | 0.00 | 0.19 | 0.961 | 0.05 | 0.22 | 0.653 |
| Meet Comfortably | -0.04 | 0.14 | 0.603 | -0.02 | 0.19 | 0.786 | 0.02 | 0.22 | 0.834 |
| Cannot meet (ref.) |  |  |  |  |  |  |  |  |  |
| **Education Plan** |  |  |  |  |  |  |  |  |  |
| Associate Degree | -0.13 | 0.22 | 0.118 | **-0.13** | **0.24** | **0.087** | **-0.15** | **0.24** | **0.034** |
| Bachelor’s Degree | -0.13 | 0.18 | 0.200 | **-0.16** | **0.20** | **0.065** | **-0.20** | **0.20** | **0.018** |
| Master’s Degree | -0.15 | 0.17 | 0.170 | **-0.20** | **0.20** | **0.035** | **-0.28** | **0.20** | **0.003** |
| Doctorate Degree | -0.14 | 0.17 | 0.189 | **-0.16** | **0.19** | **0.075** | **-0.20** | **0.19** | **0.024** |
| Certificate (ref.) |  |  |  |  |  |  |  |  |  |
| **Numeracy Level** | **0.09** | **0.02** | **0.068** | **0.08** | **0.03** | **0.091** | 0.07 | 0.03 | 0.171 |
| **Baseline Use of NETP** | **-0.15** | **0.10** | **0.006** | **-0.10** | **0.12** | **0.048** | **-0.14** | **0.12** | **0.007** |

Table: Regression models with Demographic Characteristics Predicting Perceived Risk of Using Each New and Emerging Tobacco Product at Follow Up (Continued)

|  | **Perceived risk of little cigars and cigarillos** | | | **Perceived risk of snus use** | | |
| --- | --- | --- | --- | --- | --- | --- |
|  | Beta | SE | P | Beta | SE | P |
| **Age** | 0.06 | 0.02 | 0.195 | 0.07 | 0.02 | 0.117 |
| **Being Female** | 0.06 | 0.09 | 0.240 | 0.03 | 0.08 | 0.531 |
| **Having a Child** | -0.02 | 0.15 | 0.666 | -0.05 | 0.15 | 0.378 |
|  |  |  |  |  |  |  |
| **Basic Expenses** |  |  |  |  |  |  |
| Just Meet | **-0.16** | **0.13** | **0.038** | -0.08 | 0.13 | 0.325 |
| Meet Adequately | -0.02 | 0.12 | 0.790 | 0.00 | 0.12 | 0.955 |
| Meet Comfortably | -0.10 | 0.13 | 0.180 | -0.06 | 0.13 | 0.378 |
| Cannot meet (ref.) |  |  |  |  |  |  |
| **Education Plan** |  |  |  |  |  |  |
| Associate Degree | -0.09 | 0.23 | 0.313 | -0.08 | 0.24 | 0.358 |
| Bachelor’s Degree | -0.04 | 0.19 | 0.674 | 0.00 | 0.18 | 0.968 |
| Master’s Degree | -0.03 | 0.19 | 0.762 | 0.05 | 0.18 | 0.625 |
| Doctorate Degree | -0.05 | 0.19 | 0.655 | -0.03 | 0.18 | 0.763 |
| Certificate (ref.) |  |  |  |  |  |  |
| **Numeracy Level** | 0.05 | 0.02 | 0.346 | **0.12** | **0.02** | **0.025** |
| **Baseline Use of NETP** | -0.02 | 0.11 | 0.668 | **-0.16** | **0.10** | **0.004** |
