# Supplementary material for: Mobile Text Messaging for Tobacco Risk Communication Among Young Adult Community College Students: Randomized Trial of Project Debunk
Source: JMIR Mhealth Uhealth. 2021 Nov 24;9(11):e25618. doi: 10.2196/25618 (PMC8663493; doi:10.2196/25618)
Supplement: Multimedia Appendix 4 [file mhealth_v9i11e25618_app4.docx]

**Appendix 4**

**Demographic characteristics and Risk Perception Ratings**

**Appendix 4, Table 1:** Demographic characteristics and Risk Perception Ratings

|  | **Total Sample** | **Emotional Messages Only** | **Rational Messages Only** | **Simple Messages Only** |
| --- | --- | --- | --- | --- |
| **Perceived CTP Risk at Baseline** | 2.56 (0.69) | 2.58 (0.71) | 2.55 (0.68) | 2.60 (0.66) |
| **Perceived CTP Risk at Follow Up** | 2.64 (0.71) | 2.74 (0.58) | 2.72 (0.59) | 2.75 (0.57) |
| **Perceived NETP Risk at Baseline** | 2.16 (0.77) | 2.19 ( 0.77) | 2.13 (0.76) | 2.23 (0.73) |
| **Perceived NETP Risk at Follow Up** | 2.41 (0.76) | 2.38 (0.79) | 2.43 (0.73) | 2.46 (0.76) |
| **Age, M (SD)** | 20.78 (2.18) | 20.94 (2.21) | 20.62 (2.14) | 20.80 (2.18) |
| **Being Female** | 32.55% (207/636) | 33.12% (104/314) | 31.99% (103/322) | 29.17% (91/312) |
| **Having a Child** | 9.12% (58/636) | 9.87% (31/314) | 8.39% (27/322) | 8.97% (28/312) |
| **Basic Expenses, % (n/N)** |  |  |  |  |
| Just Meet | 35.06% (223/636) | 7.32% (23/314) | 7.14% (23/322) | 8.97% (28/312) |
| Meet Adequately | 31.29% (199/636) | 32.48% (102/314) | 37.58% (121/322) | 36.22% (113/312) |
| Meet Comfortably | 26.42% (168/636) | 33.12% (104/314) | 29.5% (95/322) | 28.85% (90/312) |
| Cannot meet | 7.23% (46/636) | 27.07% (85/314) | 25.78% (83/322) | 25.96% (81/312) |
| **Education Plan, % (n/N)** |  |  |  |  |
| Associate Degree | 10.38% (66/636) | 9.55% (30/314) | 11.18% (36/322) | 9.29% (29/312) |
| Bachelor’s Degree | 24.84% (158/636) | 24.84% (78/314) | 24.84% (80/322) | 25% (78/312) |
| Master’s Degree | 30.35% (193/636) | 30.89% (97/314) | 29.81% (96/322) | 29.49% (92/312) |
| Doctorate Degree | 29.09% (185/636) | 29.62% (93/314) | 28.57% (92/322) | 31.09% (97/312) |
| Certificate | 5.35% (34/636) | 5.1% (16/314) | 5.59% (18/322) | 5.13% (16/312) |
| **Numeracy Level, M (SD)** | 4.75 (1.87) | 4.72 (1.92) | 4.77 (1.81) | 4.86 (1.96) |

**Appendix 4, Table 1:** Demographic characteristics and Risk Perception Ratings (Continued)

|  | **Complex Messages Only** | **Gain-framed Messages Only** | **Loss-framed Messages Only** |
| --- | --- | --- | --- |
| **Perceived CTP Risk at Baseline** | 2.52 (0.73) | 2.61 (0.65) | 2.51 (0.73) |
| **Perceived CTP Risk at Follow Up** | 2.70 (0.60) | 2.72 (0.59) | 2.73 (0.58) |
| **Perceived NETP Risk at Baseline** | 2.09 (0.79) | 2.24 (0.75) | 2.08 (0.78) |
| **Perceived NETP Risk at Follow Up** | 2.36 (0.77) | 2.43 (0.76) | 2.39 (0.77) |
| **Age, M (SD)** | 20.75 (2.18) | 20.82 (2.21) | 20.73 (2.15) |
| **Being Female** | 35.80% (116/324) | 28.57% (90/315) | 63.55% (204/321) |
| **Having a Child** | 9.26% (30/324) | 9.21% (29/315) | 9.03% (29/321) |
| **Basic Expenses, % (n/N)** |  |  |  |
| Just Meet | 5.56% (18/324) | 6.35% (20/315) | 8.1% (26/321) |
| Meet Adequately | 33.95% (110/324) | 35.56% (112/315) | 34.58% (111/321) |
| Meet Comfortably | 33.64% (109/324) | 32.7% (103/315) | 29.91% (96/321) |
| Cannot meet | 26.85% (87/324) | 25.4% (80/315) | 27.41% (88/321) |
| **Education Plan, % (n/N)** |  |  |  |
| Associate Degree | 11.42% (37/324) | 11.43% (36/315) | 9.35% (30/321) |
| Bachelor’s Degree | 24.69% (80/324) | 24.13% (76/315) | 25.55% (82/321) |
| Master’s Degree | 31.17% (101/324) | 27.3% (86/315) | 33.33% (107/321) |
| Doctorate Degree | 27.16% (88/324) | 31.43% (99/315) | 26.79% (86/321) |
| Certificate | 5.56% (18/324) | 5.71% (18/315) | 4.98% (16/321) |
| **Numeracy Level, M (SD)** | 4.63 (1.77) | 4.53 (1.87) | 4.95 (1.84) |
