# Supplementary material for: Mobile Text Messaging for Tobacco Risk Communication Among Young Adult Community College Students: Randomized Trial of Project Debunk
Source: JMIR Mhealth Uhealth. 2021 Nov 24;9(11):e25618. doi: 10.2196/25618 (PMC8663493; doi:10.2196/25618)
Supplement: Multimedia Appendix 5 [file mhealth_v9i11e25618_app5.docx]

**Appendix 5**

**The 95% Confidence Intervals for Each Coefficient and for All Tables**

**Appendix 5 Table 1**. Change over time in perceived risk of using new and emerging tobacco products (NETPs) for the entire sample (N=636).^a^

|  | B | SE | P | 95% Confidence Interval | |  |
| --- | --- | --- | --- | --- | --- | --- |
| Time | 0.23 | 0.06 | <0.001 | 0.11 | 0.34 |  |
| Crossover group | -0.04 | 0.06 | 0.486 | -0.16 | 0.08 |  |
| Time by crossover group | 0.02 | 0.08 | 0.787 | -0.14 | 0.19 |  |
| Age | 0.02 | 0.01 | 0.079 | 0.00 | 0.05 |  |
| Being Female | -0.02 | 0.06 | 0.776 | -0.12 | 0.09 |  |
| Having a Child | -0.05 | 0.09 | 0.549 | -0.23 | 0.12 |  |
| Basic Expenses |  |  |  |  |  |  |
| Just Meet | 0.08 | 0.11 | 0.439 | -0.12 | 0.29 |  |
| Meet Adequately | 0.14 | 0.11 | 0.187 | -0.07 | 0.35 |  |
| Meet Comfortably | 0.10 | 0.11 | 0.377 | -0.12 | 0.31 |  |
| Cannot meet (ref.) |  |  |  |  |  |  |
| Education |  |  |  |  |  |  |
| Associate Degree | -0.09 | 0.14 | 0.523 | -0.37 | 0.19 |  |
| Bachelor’s Degree | -0.14 | 0.13 | 0.279 | -0.38 | 0.11 |  |
| Master’s Degree | -0.11 | 0.13 | 0.379 | -0.36 | 0.14 |  |
| Doctorate Degree | -0.09 | 0.13 | 0.491 | -0.33 | 0.16 |  |
| Certificate (ref.) |  |  |  |  |  |  |
| Numeracy | 0.01 | 0.01 | 0.479 | -0.02 | 0.04 |  |
| Baseline Use of NETP | -0.16 | 0.06 | 0.009 | -0.27 | -0.04 |  |
| **Receive Gain-framed Messages** | 0.10 | 0.05 | 0.051 | 0.00 | 0.20 |  |
| **Receive Emotional Messages** | 0.01 | 0.05 | 0.837 | -0.09 | 0.11 |  |
| **Receive Simple Messages** | 0.13 | 0.05 | 0.012 | 0.03 | 0.23 |  |

^a^This table is based on Table 1 of the manuscript and presents 95% confidence intervals for each association.

**Appendix 5 Table 2**. Change over time in perceived risk of using conventional tobacco products (CTPs) for the entire sample (N=636).^a^

|  | B | SE | P | 95% Confidence Interval | |  |
| --- | --- | --- | --- | --- | --- | --- |
| Time | 0.13 | 0.05 | 0.008 | 0.03 | 0.23 |  |
| Crossover group | -0.04 | 0.05 | 0.45 | -0.14 | 0.06 |  |
| Time by crossover group | 0.05 | 0.07 | 0.497 | -0.09 | 0.18 |  |
| Age | 0.00 | 0.01 | 0.935 | -0.02 | 0.02 |  |
| Being Female | 0.01 | 0.05 | 0.768 | -0.08 | 0.11 |  |
| Having a Child | 0.09 | 0.08 | 0.229 | -0.06 | 0.25 |  |
| Basic Expenses |  |  |  |  |  |  |
| Just Meet | 0.08 | 0.09 | 0.375 | -0.10 | 0.26 |  |
| Meet Adequately | 0.17 | 0.09 | 0.061 | -0.01 | 0.35 |  |
| Meet Comfortably | 0.02 | 0.09 | 0.839 | -0.16 | 0.20 |  |
| Cannot meet (ref.) |  |  |  |  |  |  |
| Education |  |  |  |  |  |  |
| Associate Degree | 0.12 | 0.12 | 0.327 | -0.12 | 0.35 |  |
| Bachelor’s Degree | 0.14 | 0.11 | 0.187 | -0.07 | 0.35 |  |
| Master’s Degree | 0.18 | 0.10 | 0.082 | -0.02 | 0.39 |  |
| Doctorate Degree | 0.16 | 0.10 | 0.126 | -0.05 | 0.37 |  |
| Certificate (ref.) |  |  |  |  |  |  |
| Numeracy | 0.01 | 0.01 | 0.284 | -0.01 | 0.04 |  |
| Baseline Use of CTP | -0.10 | 0.06 | 0.099 | -0.22 | 0.02 |  |
| **Receive Gain-framed Messages** | 0.06 | 0.04 | 0.208 | -0.03 | 0.14 |  |
| **Receive Emotional Messages** | 0.02 | 0.04 | 0.572 | -0.06 | 0.11 |  |
| **Receive Simple Messages** | 0.07 | 0.04 | 0.106 | -0.02 | 0.16 |  |

^a^This table is based on Table 1 of the manuscript and presents 95% confidence intervals for each association.

**Appendix 5 Table 3**. Change over time in perceived risk of using conventional tobacco products (CTPs) for those receiving Emotional Messages.^a^

|  | B | SE | P | 95% Confidence Interval | |  |
| --- | --- | --- | --- | --- | --- | --- |
| Time | 0.17 | 0.07 | 0.010 | 0.04 | 0.31 |  |
| Crossover group | 0.02 | 0.08 | 0.761 | -0.13 | 0.17 |  |
| Time by crossover group | -0.05 | 0.10 | 0.644 | -0.24 | 0.15 |  |
| Age | -0.01 | 0.02 | 0.361 | -0.04 | 0.02 |  |
| Being Female | -0.01 | 0.07 | 0.937 | -0.15 | 0.13 |  |
| Having a Child | 0.11 | 0.11 | 0.330 | -0.11 | 0.33 |  |
| Basic Expenses |  |  |  |  |  |  |
| Just Meet | 0.25 | 0.13 | 0.057 | -0.01 | 0.51 |  |
| Meet Adequately | 0.27 | 0.13 | 0.041 | 0.01 | 0.53 |  |
| Meet Comfortably | 0.13 | 0.14 | 0.346 | -0.14 | 0.39 |  |
| Cannot meet (ref.) |  |  |  |  |  |  |
| Education |  |  |  |  |  |  |
| Associate Degree | -0.07 | 0.18 | 0.677 | -0.42 | 0.27 |  |
| Bachelor’s Degree | 0.00 | 0.16 | 0.990 | -0.31 | 0.31 |  |
| Master’s Degree | 0.06 | 0.16 | 0.708 | -0.25 | 0.37 |  |
| Doctorate Degree | -0.04 | 0.16 | 0.786 | -0.35 | 0.26 |  |
| Certificate (ref.) |  |  |  |  |  |  |
| Numeracy | 0.02 | 0.02 | 0.278 | -0.02 | 0.05 |  |
| Baseline Use of CTP | -0.16 | 0.09 | 0.076 | -0.33 | 0.02 |  |
| **Receive Gain-framed Messages** | 0.14 | 0.07 | 0.039 | 0.01 | 0.26 |  |
| **Receive Emotional Messages** |  |  |  |  |  |  |
| **Receive Simple Messages** | 0.04 | 0.06 | 0.530 | -0.09 | 0.17 |  |

^a^This table is based on Table 4 of the manuscript and presents 95% confidence intervals for each association.

**Appendix 5 Table 4.** Change over time in perceived risk of using conventional tobacco products (CTPs) for those receiving Rational Messages.^a^

| B | SE | P | 95% Confidence Interval | |  |  |
| --- | --- | --- | --- | --- | --- | --- |
| Time | 0.09 | 0.07 | 0.198 | -0.05 | 0.23 |  |
| Crossover group | -0.10 | 0.07 | 0.189 | -0.24 | 0.05 |  |
| Time by crossover group | 0.12 | 0.10 | 0.219 | -0.07 | 0.32 |  |
| Age | 0.01 | 0.01 | 0.310 | -0.01 | 0.04 |  |
| Being Female | 0.04 | 0.07 | 0.495 | -0.08 | 0.17 |  |
| Having a Child | 0.08 | 0.11 | 0.475 | -0.14 | 0.30 |  |
| Basic Expenses |  |  |  |  |  |  |
| Just Meet | -0.14 | 0.12 | 0.248 | -0.39 | 0.10 |  |
| Meet Adequately | 0.01 | 0.13 | 0.961 | -0.24 | 0.25 |  |
| Meet Comfortably | -0.13 | 0.13 | 0.306 | -0.39 | 0.12 |  |
| Cannot meet (ref.) |  |  |  |  |  |  |
| Education |  |  |  |  |  |  |
| Associate Degree | 0.31 | 0.16 | 0.050 | <0.001 | 0.62 |  |
| Bachelor’s Degree | 0.28 | 0.14 | 0.048 | 0.00 | 0.56 |  |
| Master’s Degree | 0.29 | 0.14 | 0.045 | 0.01 | 0.57 |  |
| Doctorate Degree | 0.35 | 0.14 | 0.013 | 0.07 | 0.63 |  |
| Certificate (ref.) |  |  |  |  |  |  |
| Numeracy | 0.01 | 0.02 | 0.650 | -0.03 | 0.04 |  |
| Baseline Use of CTP | -0.03 | 0.08 | 0.740 | -0.19 | 0.14 |  |
| **Receive Gain-framed Messages** | -0.03 | 0.06 | 0.637 | -0.15 | 0.09 |  |
| **Receive Emotional Messages** |  |  |  |  |  |  |
| **Receive Simple Messages** | 0.09 | 0.06 | 0.114 | -0.02 | 0.21 |  |

^a^This table is based on Table 5 of the manuscript and presents 95% confidence intervals for each association.

**Appendix 5 Table 5**. Change over time in perceived risk of using conventional tobacco products (CTPs) for those receiving Complex Messages.^a^

|  | B | SE | P | 95% Confidence Interval | |  |
| --- | --- | --- | --- | --- | --- | --- |
| Time | 0.16 | 0.07 | 0.026 | 0.02 | 0.29 |  |
| Crossover group | -0.09 | 0.08 | 0.237 | -0.24 | 0.06 |  |
| Time by crossover group | 0.03 | 0.10 | 0.761 | -0.17 | 0.23 |  |
| Age | -0.01 | 0.02 | 0.663 | -0.04 | 0.02 |  |
| Being Female | 0.05 | 0.07 | 0.453 | -0.08 | 0.19 |  |
| Having a Child | 0.04 | 0.12 | 0.709 | -0.18 | 0.27 |  |
| Basic Expenses |  |  |  |  |  |  |
| Just Meet | 0.12 | 0.15 | 0.430 | -0.18 | 0.41 |  |
| Meet Adequately | 0.24 | 0.15 | 0.113 | -0.06 | 0.53 |  |
| Meet Comfortably | 0.11 | 0.15 | 0.472 | -0.19 | 0.41 |  |
| Cannot meet (ref.) |  |  |  |  |  |  |
| Education |  |  |  |  |  |  |
| Associate Degree | 0.19 | 0.17 | 0.269 | -0.15 | 0.53 |  |
| Bachelor’s Degree | 0.06 | 0.16 | 0.708 | -0.25 | 0.36 |  |
| Master’s Degree | 0.08 | 0.15 | 0.591 | -0.22 | 0.39 |  |
| Doctorate Degree | 0.10 | 0.16 | 0.523 | -0.21 | 0.41 |  |
| Certificate (ref.) |  |  |  |  |  |  |
| Numeracy | -0.02 | 0.02 | 0.314 | -0.06 | 0.02 |  |
| Baseline Use of CTP | 0.07 | 0.09 | 0.442 | -0.11 | 0.25 |  |
| **Receive Gain-framed Messages** | 0.04 | 0.07 | 0.571 | -0.09 | 0.17 |  |
| **Receive Emotional Messages** | 0.06 | 0.07 | 0.324 | -0.06 | 0.19 |  |
| **Receive Simple Messages** |  |  |  |  |  |  |

^a^This table is based on Table 4 of the manuscript and presents 95% confidence intervals for each association.

**Appendix 5 Table 6**. Change over time in perceived risk of using conventional tobacco products (CTPs) for those receiving Simple Messages.^a^

| B | SE | P | 95% Confidence Interval | |  |  |
| --- | --- | --- | --- | --- | --- | --- |
| Time | 0.11 | 0.07 | 0.117 | -0.03 | 0.24 |  |
| Crossover group | 0.02 | 0.07 | 0.809 | -0.12 | 0.15 |  |
| Time by crossover group | 0.05 | 0.10 | 0.620 | -0.14 | 0.24 |  |
| Age | 0.01 | 0.01 | 0.480 | -0.02 | 0.04 |  |
| Being Female | -0.02 | 0.06 | 0.808 | -0.14 | 0.11 |  |
| Having a Child | 0.19 | 0.10 | 0.072 | -0.02 | 0.39 |  |
| Basic Expenses |  |  |  |  |  |  |
| Just Meet | 0.06 | 0.11 | 0.581 | -0.15 | 0.27 |  |
| Meet Adequately | 0.10 | 0.11 | 0.356 | -0.12 | 0.32 |  |
| Meet Comfortably | -0.05 | 0.11 | 0.673 | -0.27 | 0.18 |  |
| Cannot meet (ref.) |  |  |  |  |  |  |
| Education |  |  |  |  |  |  |
| Associate Degree | 0.00 | 0.16 | 0.997 | -0.31 | 0.31 |  |
| Bachelor’s Degree | 0.24 | 0.14 | 0.088 | -0.04 | 0.51 |  |
| Master’s Degree | 0.32 | 0.14 | 0.020 | 0.05 | 0.59 |  |
| Doctorate Degree | 0.24 | 0.14 | 0.079 | -0.03 | 0.51 |  |
| Certificate (ref.) |  |  |  |  |  |  |
| Numeracy | 0.04 | 0.01 | 0.004 | 0.01 | 0.07 |  |
| Baseline Use of CTP | -0.30 | 0.08 | 0.000 | -0.46 | -0.15 |  |
| **Receive Gain-framed Messages** | 0.10 | 0.06 | 0.090 | -0.02 | 0.21 |  |
| **Receive Emotional Messages** | -0.01 | 0.06 | 0.889 | -0.12 | 0.10 |  |
| **Receive Simple Messages** |  |  |  |  |  |  |

^a^This table is based on Table 5 of the manuscript and presents 95% confidence intervals for each association.

**Appendix 5 Table 7**. Change over time in perceived risk of using conventional tobacco products (CTPs) for those receiving Loss Messages.^a^

|  | B | SE | P | 95% Confidence Interval | |  |
| --- | --- | --- | --- | --- | --- | --- |
| Time | 0.18 | 0.07 | 0.012 | 0.04 | 0.32 |  |
| Crossover group | -0.01 | 0.08 | 0.940 | -0.16 | 0.14 |  |
| Time by crossover group | 0.06 | 0.10 | 0.543 | -0.14 | 0.26 |  |
| Age | -0.01 | 0.02 | 0.628 | -0.04 | 0.02 |  |
| Being Female | 0.04 | 0.07 | 0.545 | -0.09 | 0.18 |  |
| Having a Child | 0.19 | 0.12 | 0.092 | -0.03 | 0.42 |  |
| Basic Expenses |  |  |  |  |  |  |
| Just Meet | 0.20 | 0.13 | 0.106 | -0.04 | 0.45 |  |
| Meet Adequately | 0.23 | 0.13 | 0.073 | -0.02 | 0.48 |  |
| Meet Comfortably | 0.08 | 0.13 | 0.552 | -0.18 | 0.33 |  |
| Cannot meet (ref.) |  |  |  |  |  |  |
| Education |  |  |  |  |  |  |
| Associate Degree | 0.05 | 0.18 | 0.803 | -0.31 | 0.40 |  |
| Bachelor’s Degree | 0.00 | 0.16 | 0.992 | -0.32 | 0.31 |  |
| Master’s Degree | 0.01 | 0.16 | 0.957 | -0.30 | 0.32 |  |
| Doctorate Degree | -0.04 | 0.16 | 0.809 | -0.35 | 0.28 |  |
| Certificate (ref.) |  |  |  |  |  |  |
| Numeracy | 0.01 | 0.02 | 0.519 | -0.02 | 0.05 |  |
| Baseline Use of CTP | -0.14 | 0.09 | 0.131 | -0.32 | 0.04 |  |
| Receive Gain-framed Messages |  |  |  |  |  |  |
| Receive Emotional Messages | -0.05 | 0.06 | 0.468 | -0.17 | 0.08 |  |
| Receive Simple Messages | 0.07 | 0.06 | 0.311 | -0.06 | 0.19 |  |

^a^This table is based on Table 4 of the manuscript and presents 95% confidence intervals for each association.

**Appendix 5 Table 8**. Change over time in perceived risk of using conventional tobacco products (CTPs) for those receiving Gain Messages.^a^

| B | SE | P | 95% Confidence Interval | |  |  |
| --- | --- | --- | --- | --- | --- | --- |
| Time | 0.08 | 0.07 | 0.226 | -0.05 | 0.21 |  |
| Crossover group | -0.06 | 0.07 | 0.414 | -0.20 | 0.08 |  |
| Time by crossover group | 0.03 | 0.10 | 0.749 | -0.16 | 0.22 |  |
| Age | 0.01 | 0.01 | 0.647 | -0.02 | 0.03 |  |
| Being Female | 0.00 | 0.07 | 0.982 | -0.13 | 0.13 |  |
| Having a Child | 0.02 | 0.11 | 0.841 | -0.19 | 0.23 |  |
| Basic Expenses |  |  |  |  |  |  |
| Just Meet | -0.12 | 0.13 | 0.356 | -0.39 | 0.14 |  |
| Meet Adequately | 0.04 | 0.13 | 0.777 | -0.23 | 0.30 |  |
| Meet Comfortably | -0.11 | 0.14 | 0.419 | -0.39 | 0.16 |  |
| Cannot meet (ref.) |  |  |  |  |  |  |
| Education |  |  |  |  |  |  |
| Associate Degree | 0.18 | 0.16 | 0.261 | -0.13 | 0.49 |  |
| Bachelor’s Degree | 0.24 | 0.14 | 0.095 | -0.04 | 0.52 |  |
| Master’s Degree | 0.32 | 0.14 | 0.024 | 0.04 | 0.60 |  |
| Doctorate Degree | 0.32 | 0.14 | 0.021 | 0.05 | 0.59 |  |
| Certificate (ref.) |  |  |  |  |  |  |
| Numeracy | 0.01 | 0.02 | 0.372 | -0.02 | 0.05 |  |
| Baseline Use of CTP | -0.05 | 0.08 | 0.514 | -0.21 | 0.11 |  |
| Receive Gain-framed Messages |  |  |  |  |  |  |
| Receive Emotional Messages | 0.09 | 0.06 | 0.141 | -0.03 | 0.21 |  |
| Receive Simple Messages | 0.06 | 0.06 | 0.357 | -0.06 | 0.17 |  |

^a^This table is based on Table 5 of the manuscript and presents 95% confidence intervals for each association.

**Appendix 5 Table 9**. Change over time in perceived risk of using new and emerging tobacco products (NETPs) for those receiving Emotional Messages.^a^

|  | B | SE | P | 95% Confidence Interval | |  |
| --- | --- | --- | --- | --- | --- | --- |
| Time | 0.24 | 0.09 | 0.006 | 0.07 | 0.41 |  |
| Crossover group | 0.03 | 0.09 | 0.719 | -0.14 | 0.21 |  |
| Time by crossover group | -0.11 | 0.13 | 0.41 | -0.36 | 0.14 |  |
| Age | 0.02 | 0.02 | 0.227 | -0.01 | 0.06 |  |
| Being Female | -0.12 | 0.08 | 0.14 | -0.28 | 0.04 |  |
| Having a Child | 0.04 | 0.13 | 0.759 | -0.21 | 0.29 |  |
| Basic Expenses |  |  |  |  |  |  |
| Just Meet | 0.07 | 0.15 | 0.662 | -0.23 | 0.36 |  |
| Meet Adequately | 0.08 | 0.15 | 0.586 | -0.22 | 0.38 |  |
| Meet Comfortably | 0.09 | 0.16 | 0.554 | -0.21 | 0.40 |  |
| Cannot meet (ref.) |  |  |  |  |  |  |
| Education |  |  |  |  |  |  |
| Associate Degree | -0.25 | 0.21 | 0.229 | -0.65 | 0.16 |  |
| Bachelor’s Degree | -0.26 | 0.19 | 0.168 | -0.62 | 0.11 |  |
| Master’s Degree | -0.23 | 0.19 | 0.205 | -0.60 | 0.13 |  |
| Doctorate Degree | -0.23 | 0.18 | 0.219 | -0.59 | 0.13 |  |
| Certificate (ref.) |  |  |  |  |  |  |
| Numeracy | 0.03 | 0.02 | 0.151 | -0.01 | 0.07 |  |
| Baseline Use of NETP | -0.22 | 0.09 | 0.009 | -0.39 | -0.05 |  |
| Receive Gain-framed Messages | 0.16 | 0.07 | 0.036 | 0.01 | 0.30 |  |
| Receive Emotional Messages |  |  |  |  |  |  |
| Receive Simple Messages | 0.06 | 0.07 | 0.412 | -0.08 | 0.21 |  |

^a^This table is based on Table 2 of the manuscript and presents 95% confidence intervals for each association.

**Appendix 5 Table 10**. Change over time in perceived risk of using new and emerging tobacco products (NETPs) for those receiving Rational Messages.^a^

| B | SE | P | 95% Confidence Interval | |  |  |
| --- | --- | --- | --- | --- | --- | --- |
| Time | 0.22 | 0.08 | 0.005 | 0.07 | 0.37 |  |
| Crossover group | -0.12 | 0.09 | 0.164 | -0.29 | 0.05 |  |
| Time by crossover group | 0.14 | 0.11 | 0.213 | -0.08 | 0.36 |  |
| Age | 0.03 | 0.02 | 0.160 | -0.01 | 0.06 |  |
| Being Female | 0.07 | 0.08 | 0.363 | -0.08 | 0.22 |  |
| Having a Child | -0.16 | 0.13 | 0.217 | -0.43 | 0.10 |  |
| Basic Expenses |  |  |  |  |  |  |
| Just Meet | 0.10 | 0.15 | 0.523 | -0.20 | 0.39 |  |
| Meet Adequately | 0.16 | 0.15 | 0.280 | -0.13 | 0.46 |  |
| Meet Comfortably | 0.09 | 0.16 | 0.579 | -0.22 | 0.39 |  |
| Cannot meet (ref.) |  |  |  |  |  |  |
| Education |  |  |  |  |  |  |
| Associate Degree | 0.04 | 0.20 | 0.824 | -0.34 | 0.43 |  |
| Bachelor’s Degree | -0.03 | 0.18 | 0.882 | -0.37 | 0.32 |  |
| Master’s Degree | 0.00 | 0.18 | 0.994 | -0.35 | 0.34 |  |
| Doctorate Degree | 0.04 | 0.17 | 0.801 | -0.30 | 0.38 |  |
| Certificate (ref.) |  |  |  |  |  |  |
| Numeracy | 0.00 | 0.02 | 0.859 | -0.04 | 0.04 |  |
| Baseline Use of NETP | -0.09 | 0.09 | 0.314 | -0.26 | 0.08 |  |
| Receive Gain-framed Messages | 0.07 | 0.07 | 0.316 | -0.07 | 0.22 |  |
| Receive Emotional Messages |  |  |  |  |  |  |
| Receive Simple Messages | 0.21 | 0.07 | 0.003 | 0.07 | 0.35 |  |

^a^This table is based on Table 3 of the manuscript and presents 95% confidence intervals for each association.

**Appendix 5 Table 11**. Change over time in perceived risk of using new and emerging tobacco products (NETPs) for those receiving Complex Messages.^a^

|  | B | SE | P | 95% Confidence Interval | |  |
| --- | --- | --- | --- | --- | --- | --- |
| Time | 0.21 | 0.08 | 0.011 | 0.05 | 0.37 |  |
| Crossover group | -0.09 | 0.09 | 0.324 | -0.26 | 0.09 |  |
| Time by crossover group | 0.11 | 0.12 | 0.342 | -0.12 | 0.35 |  |
| Age | 0.02 | 0.02 | 0.192 | -0.01 | 0.06 |  |
| Being Female | 0.04 | 0.08 | 0.614 | -0.11 | 0.19 |  |
| Having a Child | -0.22 | 0.13 | 0.092 | -0.47 | 0.04 |  |
| Basic Expenses |  |  |  |  |  |  |
| Just Meet | 0.06 | 0.17 | 0.706 | -0.26 | 0.39 |  |
| Meet Adequately | 0.11 | 0.17 | 0.507 | -0.22 | 0.44 |  |
| Meet Comfortably | 0.04 | 0.17 | 0.839 | -0.30 | 0.37 |  |
| Cannot meet (ref.) |  |  |  |  |  |  |
| Education |  |  |  |  |  |  |
| Associate Degree | -0.04 | 0.20 | 0.844 | -0.43 | 0.35 |  |
| Bachelor’s Degree | -0.22 | 0.18 | 0.220 | -0.57 | 0.13 |  |
| Master’s Degree | -0.22 | 0.18 | 0.221 | -0.57 | 0.13 |  |
| Doctorate Degree | -0.19 | 0.18 | 0.281 | -0.55 | 0.16 |  |
| Certificate (ref.) |  |  |  |  |  |  |
| Numeracy | -0.02 | 0.02 | 0.356 | -0.06 | 0.02 |  |
| Baseline Use of NETP | -0.16 | 0.09 | 0.060 | -0.33 | 0.01 |  |
| Receive Gain-framed Messages | 0.13 | 0.07 | 0.081 | -0.02 | 0.27 |  |
| Receive Emotional Messages | 0.09 | 0.07 | 0.197 | -0.05 | 0.24 |  |
| Receive Simple Messages |  |  |  |  |  |  |

^a^This table is based on Table 2 of the manuscript and presents 95% confidence intervals for each association.

**Appendix 5 Table 12**. Change over time in perceived risk of using new and emerging tobacco products (NETPs) for those receiving Simple Messages.^a^

| B | SE | P | 95% Confidence Interval | |  |  |
| --- | --- | --- | --- | --- | --- | --- |
| Time | 0.25 | 0.08 | 0.003 | 0.09 | 0.42 |  |
| Crossover group | -0.02 | 0.09 | 0.852 | -0.18 | 0.15 |  |
| Time by crossover group | -0.08 | 0.12 | 0.498 | -0.32 | 0.16 |  |
| Age | 0.02 | 0.02 | 0.268 | -0.01 | 0.05 |  |
| Being Female | -0.07 | 0.08 | 0.395 | -0.23 | 0.09 |  |
| Having a Child | 0.12 | 0.13 | 0.333 | -0.13 | 0.38 |  |
| Basic Expenses |  |  |  |  |  |  |
| Just Meet | 0.10 | 0.14 | 0.446 | -0.16 | 0.37 |  |
| Meet Adequately | 0.15 | 0.14 | 0.285 | -0.12 | 0.42 |  |
| Meet Comfortably | 0.16 | 0.14 | 0.268 | -0.12 | 0.44 |  |
| Cannot meet (ref.) |  |  |  |  |  |  |
| Education |  |  |  |  |  |  |
| Associate Degree | -0.17 | 0.20 | 0.393 | -0.57 | 0.22 |  |
| Bachelor’s Degree | -0.04 | 0.18 | 0.827 | -0.39 | 0.31 |  |
| Master’s Degree | 0.03 | 0.18 | 0.872 | -0.32 | 0.37 |  |
| Doctorate Degree | 0.03 | 0.17 | 0.872 | -0.31 | 0.37 |  |
| Certificate (ref.) |  |  |  |  |  |  |
| Numeracy | 0.04 | 0.02 | 0.042 | 0.00 | 0.07 |  |
| Baseline Use of NETP | -0.16 | 0.09 | 0.064 | -0.33 | 0.01 |  |
| Receive Gain-framed Messages | 0.09 | 0.07 | 0.223 | -0.05 | 0.23 |  |
| Receive Emotional Messages | -0.07 | 0.07 | 0.336 | -0.21 | 0.07 |  |
| Receive Simple Messages |  |  |  |  |  |  |

^a^This table is based on Table 3 of the manuscript and presents 95% confidence intervals for each association.

**Appendix 5 Table 13**. Change over time in perceived risk of using new and emerging tobacco products (NETPs) for those receiving Loss Messages.^a^

|  | B | SE | P | 95% Confidence Interval | |  |
| --- | --- | --- | --- | --- | --- | --- |
| Time | 0.20 | 0.08 | 0.014 | 0.04 | 0.36 |  |
| Crossover group | -0.04 | 0.09 | 0.630 | -0.21 | 0.13 |  |
| Time by crossover group | 0.19 | 0.12 | 0.107 | -0.04 | 0.43 |  |
| Age | 0.02 | 0.02 | 0.176 | -0.01 | 0.06 |  |
| Being Female | 0.08 | 0.08 | 0.294 | -0.07 | 0.24 |  |
| Having a Child | 0.16 | 0.13 | 0.229 | -0.10 | 0.41 |  |
| Basic Expenses |  |  |  |  |  |  |
| Just Meet | 0.09 | 0.14 | 0.541 | -0.19 | 0.37 |  |
| Meet Adequately | 0.04 | 0.14 | 0.785 | -0.24 | 0.32 |  |
| Meet Comfortably | 0.05 | 0.15 | 0.725 | -0.24 | 0.34 |  |
| Cannot meet (ref.) |  |  |  |  |  |  |
| Education |  |  |  |  |  |  |
| Associate Degree | -0.29 | 0.21 | 0.169 | -0.70 | 0.12 |  |
| Bachelor’s Degree | -0.22 | 0.19 | 0.242 | -0.59 | 0.15 |  |
| Master’s Degree | -0.26 | 0.19 | 0.155 | -0.63 | 0.10 |  |
| Doctorate Degree | -0.25 | 0.19 | 0.180 | -0.62 | 0.12 |  |
| Certificate (ref.) |  |  |  |  |  |  |
| Numeracy | -0.02 | 0.02 | 0.432 | -0.06 | 0.02 |  |
| Baseline Use of NETP | -0.22 | 0.08 | 0.010 | -0.38 | -0.05 |  |
| Receive Gain-framed Messages |  |  |  |  |  |  |
| Receive Emotional Messages | -0.02 | 0.07 | 0.777 | -0.17 | 0.12 |  |
| Receive Simple Messages | 0.15 | 0.07 | 0.041 | 0.01 | 0.30 |  |

^a^This table is based on Table 2 of the manuscript and presents 95% confidence intervals for each association.

**Appendix 5 Table 14**. Change over time in perceived risk of using new and emerging tobacco products (NETPs) for those receiving Gain Messages.^a^

| B | SE | P | 95% Confidence Interval | |  |  |
| --- | --- | --- | --- | --- | --- | --- |
| Time | 0.25 | 0.08 | 0.002 | 0.09 | 0.41 |  |
| Crossover group | -0.04 | 0.09 | 0.648 | -0.21 | 0.13 |  |
| Time by crossover group | -0.14 | 0.12 | 0.236 | -0.38 | 0.09 |  |
| Age | 0.02 | 0.02 | 0.173 | -0.01 | 0.06 |  |
| Being Female | -0.10 | 0.08 | 0.215 | -0.25 | 0.06 |  |
| Having a Child | -0.21 | 0.13 | 0.097 | -0.46 | 0.04 |  |
| Basic Expenses |  |  |  |  |  |  |
| Just Meet | 0.04 | 0.16 | 0.801 | -0.27 | 0.35 |  |
| Meet Adequately | 0.20 | 0.16 | 0.219 | -0.12 | 0.51 |  |
| Meet Comfortably | 0.13 | 0.16 | 0.435 | -0.19 | 0.45 |  |
| Cannot meet (ref.) |  |  |  |  |  |  |
| Education |  |  |  |  |  |  |
| Associate Degree | 0.07 | 0.19 | 0.707 | -0.30 | 0.45 |  |
| Bachelor’s Degree | -0.09 | 0.17 | 0.598 | -0.43 | 0.25 |  |
| Master’s Degree | -0.01 | 0.17 | 0.957 | -0.35 | 0.33 |  |
| Doctorate Degree | 0.04 | 0.17 | 0.822 | -0.29 | 0.37 |  |
| Certificate (ref.) |  |  |  |  |  |  |
| Numeracy | 0.03 | 0.02 | 0.133 | -0.01 | 0.07 |  |
| Baseline Use of NETP | -0.10 | 0.09 | 0.259 | -0.27 | 0.07 |  |
| Receive Gain-framed Messages |  |  |  |  |  |  |
| Receive Emotional Messages | 0.03 | 0.07 | 0.670 | -0.11 | 0.17 |  |
| Receive Simple Messages | 0.07 | 0.07 | 0.301 | -0.07 | 0.21 |  |

^a^This table is based on Table 3 of the manuscript and presents 95% confidence intervals for each association.
